# Octopamine receptors in *Drosophila* larvae: expression, development, and behavior

**DOI:** 10.3389/fnmol.2026.1876842

**Published:** 2026-09-03

**Authors:** Alexandra Großjohann, Vincent Richter, Franziska Reinhardt, Marvin Hahmann, Ronja Badelt, Juliane Kinnigkeit, Jana Breitfeld, Peter Kovacs, Peter F. Stadler, Irene Coin, Andreas S. Thum

**Affiliations:** 1 Department of Genetics, Institute of Biology, Leipzig University, Leipzig, Germany; 2 Department of Bioinformatics, Institute of Computer Science, Interdisciplinary Center of Bioinformatics, Leipzig University, Leipzig, Germany; 3 Department of Medicine III, Division of Endocrinology, Nephrology and Rheumatology, University of Leipzig, Leipzig, Germany; 4 German Center for Diabetes Research (DZD), Neuherberg, Germany; 5 Max Planck Institute for Mathematics in the Sciences, Leipzig, Germany; 6 Department of Theoretical Chemistry, University of Vienna, Vienna, Austria; 7 Facultad de Ciencias, Universidad Nacional de Colombia, Bogotá, Colombia; 8 Santa Fe Institute, Santa Fe, NM, United States; 9 Center for Non-coding RNA in Technology and Health, University of Copenhagen, Frederiksberg, Denmark; 10 Faculty of Life Science, Institute of Biochemistry, Leipzig University, Leipzig, Germany; 11 German Centre for Integrative Biodiversity Research (iDiv) Halle-Jena-Leipzig, Leipzig, Germany

**Keywords:** biogenic amines, bulk RNA-seq analysis, feeding, neuromodulation, Trojan Gal4

## Abstract

Octopamine is involved in a variety of physiological and behavioral mechanisms throughout the *Drosophila melanogaster* life cycle. Octopaminergic neurons in both the central and the peripheral nervous system target a multitude of neurons and even non-neuronal tissues, making it challenging to analyze individual mechanisms of octopamine function. One approach to deconstructing this complex system is to examine the postsynaptic components of signal transmission, the six distinct G-protein-coupled octopamine receptors. Here, we compiled and validated a complete set of potential mutant lines of all known octopamine receptors (Oamb, Octα2R, Octβ1R, Octβ2R, Octβ3R, and Oct-TyrR) that were generated using the same genetic tool: the recently established Trojan Exon system. It integrates the Gal4/UAS binary expression strategy while simultaneously impairing receptor function. We generated a comprehensive anatomical map of receptor expression in the larva and, at the same time, analyzed the function of individual octopamine receptors during larval development, chemosensory perception, and locomotion. All octopamine receptors are expressed in the central and peripheral nervous system. Octβ2R stands out with a pronounced expression in the somatic muscles. We also observed a previously undescribed role of Octβ1R, Octβ3R, and Oct-TyrR in larval hatching and in the survival of larvae and pupae. Combined with tissue- and stage-specific gene expression data, this validated set of transgenic lines provides a basis for further functional studies of the octopaminergic system.

## Introduction

Octopamine (OA) is a biogenic amine that acts as a neuromodulator, neurotransmitter, and neurohormone in invertebrates and plays a vital role in regulating a variety of behavioral and physiological processes. Extensive research has been conducted on its functions in a range of species, including *Drosophila melanogaster*, *Apis mellifera*, and *Caenorhabditis elegans*, as well as on its structural analog norepinephrine in vertebrates (reviewed in David and Coulon, 1985; Roeder, 2005; Farooqui, 2012; Roeder, 2020; Mezheritskiy et al., 2024).

**FIGURE 1:**
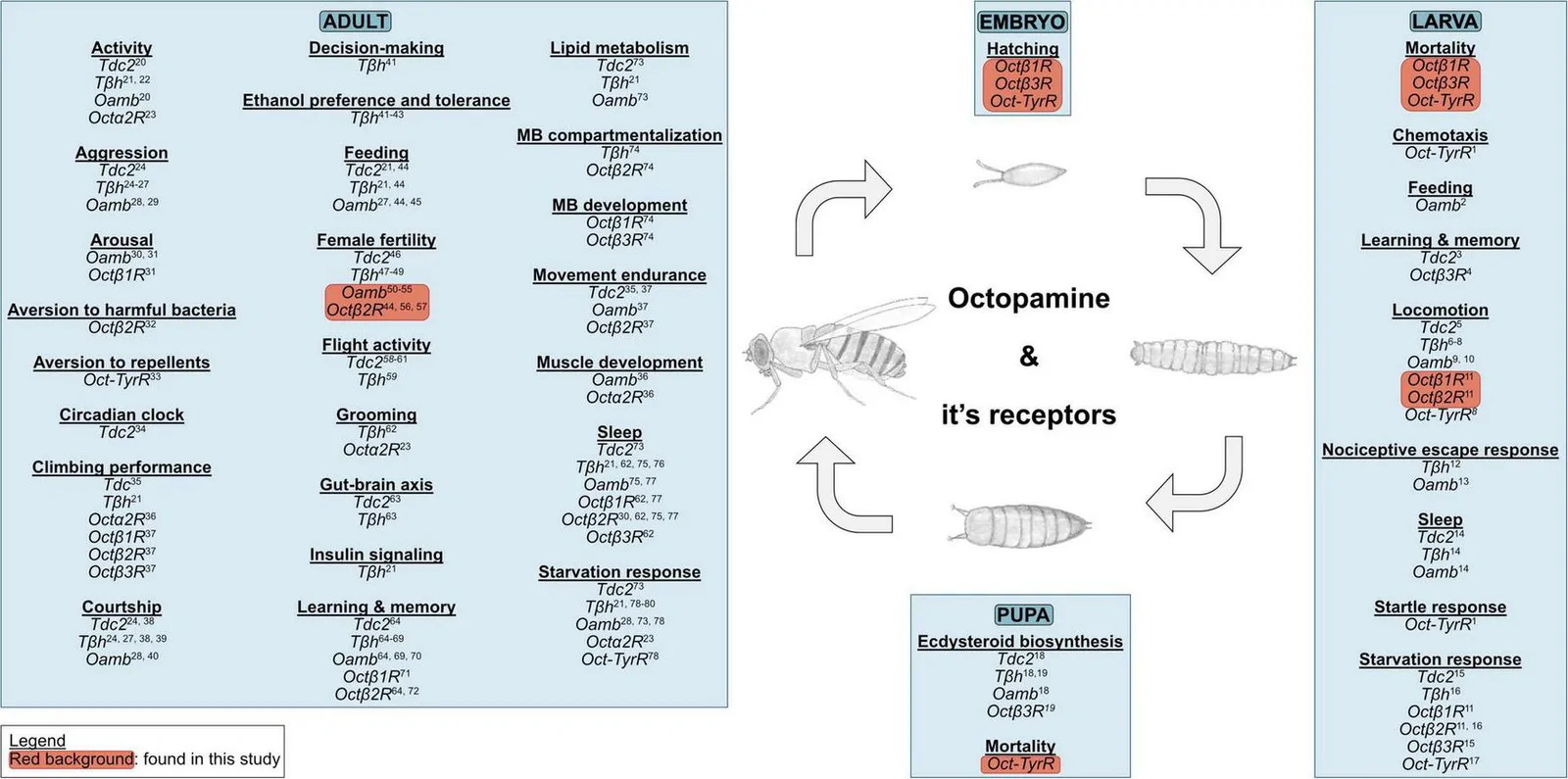
Overview of OAR functions from embryo to adult. Four developmental stages are distinguished: embryo, third-instar larva, pupa, and adult. This summary includes novel functions from this study (highlighted in red) and earlier published functions: 1 (Ma et al., 2016); 2 (Branch et al., 2017); 3 (Selcho et al., 2014); 4 (Franke et al., 2026); 5 (Pauls et al., 2015); 6 (Saraswati et al., 2004); 7 (Fox et al., 2006); 8 (Selcho et al., 2012); 9 (Bakshinska et al., 2025); 10 (Blaum et al., 2025); 11 (Koon and Budnik, 2012); 12 (Li et al., 2023); 13 (Boivin et al., 2026); 14 (Szuperak et al., 2018); 15 (Zhang et al., 2013); 16 (Koon et al., 2011); 17 (Schützler et al., 2019); 18 (Imura et al., 2020); 19 (Ohhara et al., 2015); 20 (Pauls et al., 2021); 21 (Li et al., 2016); 22 (Bragina et al., 2025); 23 (Nakagawa et al., 2022); 24 (Hoyer et al., 2008); 25 (Baier et al., 2002); 26 (Zhou et al., 2008); 27 (Andrews et al., 2014); 28 (Luo et al., 2014); 29 (Watanabe et al., 2017); 30 (Crocker et al., 2010); 31 (Kula-Eversole et al., 2010); 32 (Wang et al., 2026); 33 (Kutsukake et al., 2000); 34 (Huang et al., 2013); 35 (Sujkowski and Wessells, 2018); 36 (El-Kholy et al., 2022); 37 (Sujkowski et al., 2017); 38 (Certel et al., 2010); 39 (Certel et al., 2007); 40 (Zhou et al., 2012); 41 (Classen and Scholz, 2018); 42 (Schneider et al., 2012); 43 (Scholz et al., 2000); 44 (Youn et al., 2018); 45 (Cole et al., 2005); 46 (Monastirioti et al., 1996); 47 (Monastirioti, 2003); 48 (Rezaval et al., 2014); 49 (Lee et al., 2003); 50 (Lee et al., 2009); 51 (Avila et al., 2012); 52 (Deady and Sun, 2015); 53 (Yoshinari et al., 2020); 54 (Rohrbach et al., 2024); 55 (Lim et al., 2014); 56 (Li et al., 2015); 57 (Wang et al., 2016); 58 (Manjila et al., 2019); 59 (Brembs et al., 2007); 60 (Suver et al., 2012); 61 (van Breugel et al., 2014); 62 (Zhao et al., 2021); 63 (Schretter et al., 2018); 64 (Burke et al., 2012); 65 (Schwaerzel et al., 2003); 66 (Sitaraman et al., 2010); 67 (Yarali and Gerber, 2010); 68 (Berger et al., 2024); 69 (Huetteroth et al., 2015); 70 (Kim et al., 2013); 71 (Sabandal et al., 2020); 72 (Yang et al., 2016); 73 (Erion et al., 2012); 74 (Wu et al., 2025); 75 (Deng et al., 2019); 76 (Crocker and Sehgal, 2008); 77 (Reyes et al., 2026); 78 (Damrau et al., 2018); 79 (Yang et al., 2015); 80 (Yu et al., 2016).

In *Drosophila*, octopaminergic cells that produce OA via Tyramine β-hydroxylase (Tβh), are found not only in the central and peripheral nervous system of both adults (Monastirioti et al., 1995; Monastirioti, 2003; Zhou et al., 2008; Busch et al., 2009; Busch and Tanimoto, 2010; Schneider et al., 2012; Pauls et al., 2018; Babski et al., 2024; Selcho, 2024) and larvae (Monastirioti et al., 1995; Monastirioti et al., 1996; Koon et al., 2011; Selcho et al., 2012; Selcho et al., 2014), but also in the adult female reproductive system (Monastirioti et al., 1996; Monastirioti, 2003; Rezaval et al., 2014). OA is versatile in its function, being involved in activity, arousal, aversion to harmful bacteria, aversion to repellents, the circadian clock, climbing activity, courtship behavior, decision-making, female fertility, ethanol preference and tolerance, feeding, flight activity, grooming, gut-brain axis, lipid metabolism, male aggression, mushroom body development, memory formation, movement endurance, muscle development, sleep, starvation response (Figure 1; Monastirioti et al., 1996; Kutsukake et al., 2000; Scholz et al., 2000; Baier et al., 2002; Lee et al., 2003; Monastirioti, 2003; Schwaerzel et al., 2003; Cole et al., 2005; Brembs et al., 2007; Certel et al., 2007; Crocker and Sehgal, 2008; Hoyer et al., 2008; Zhou et al., 2008; Lee et al., 2009; Certel et al., 2010; Crocker et al., 2010; Kula-Eversole et al., 2010; Sitaraman et al., 2010; Yarali and Gerber, 2010; Avila et al., 2012; Burke et al., 2012; Erion et al., 2012; Schneider et al., 2012; Suver et al., 2012; Zhou et al., 2012; Huang et al., 2013; Kim et al., 2013; Andrews et al., 2014; Lim et al., 2014; Luo et al., 2014; Rezaval et al., 2014; van Breugel et al., 2014; Deady and Sun, 2015; Huetteroth et al., 2015; Li et al., 2015; Yang et al., 2015; Li et al., 2016; Wang et al., 2016; Yang et al., 2016; Yu et al., 2016; Sujkowski et al., 2017; Watanabe et al., 2017; Classen and Scholz, 2018; Damrau et al., 2018; Schretter et al., 2018; Sujkowski and Wessells, 2018; Youn et al., 2018; Deng et al., 2019; Manjila et al., 2019; Sabandal et al., 2020; Yoshinari et al., 2020; Pauls et al., 2021; Zhao et al., 2021; El-Kholy et al., 2022; Nakagawa et al., 2022; Berger et al., 2024; Rohrbach et al., 2024; Bragina et al., 2025; Wu et al., 2025; Reyes et al., 2026; Wang et al., 2026). In larvae, OA plays a role in chemotaxis, feeding, learning and memory, locomotion, nociceptive escape behavior, sleep, startle response, starvation response (Saraswati et al., 2004; Fox et al., 2006; Koon et al., 2011; Koon and Budnik, 2012; Selcho et al., 2012; Zhang et al., 2013; Selcho et al., 2014; Pauls et al., 2015; Ma et al., 2016; Branch et al., 2017; Szuperak et al., 2018; Schützler et al., 2019; Li et al., 2023; Bakshinska et al., 2025; Blaum et al., 2025; Boivin et al., 2026; Franke et al., 2026). In contrast to behavior, the involvement of OA in early development, ranging from the embryo to the pupa, has been scarcely investigated.

OA can bind to six distinct G-protein-coupled receptors (GPCRs) in *Drosophila*, which are classified into three groups: (1) α-adrenergic-like receptors, including Oamb and Octα2R; (2) β-adrenergic-like receptors, consisting of Octβ1R, Octβ2R, and Octβ3R; and (3) a tyramine (TA) type 1 receptor, known as Oct-TyrR (Table 1). Each receptor class differs in its structure, with its classification derived from the vertebrate adrenergic receptors. Upon OA binding, different G-proteins interact with the receptors, inducing different downstream signals (Table 1). Oamb activation increases Ca^2+^ levels via the Gαq-mediated phospholipase C pathway (Han et al., 1998; Balfanz et al., 2005). Interestingly, depending on the ligand concentration, the Ca^2+^ response can be a single event that slowly declines (high concentration) or oscillatory (low concentration; Balfanz et al., 2005). A Ca^2+^/Calmodulin-dependent protein kinase II activity (CaMKII) in the oviduct epithelium has also been reported (Lee et al., 2009). The second α-adrenergic-like receptor, Octα2R, inhibits the adenylyl cyclase pathway via Gαi/o, leading to a decrease in cyclic adenosine monophosphate (cAMP) level (Qi et al., 2017). All three β-adrenergic-like receptors have been found to interact with the Gαs protein, which leads to an activation of the adenylyl cyclase pathway and an increase in cAMP (Octβ1R: Maqueira et al., 2005; Balfanz et al., 2005; Octβ2R and Octβ3R: Maqueira et al., 2005). Yet for Octβ1R, conflicting evidence suggests that it may reduce cAMP levels by coupling to the inhibitory Gαi/o (Koon and Budnik, 2012). The Oct-Tyr receptor can be activated by two ligands, OA and TA, resulting in two possible downstream signaling cascades: either Gαq activity leads to a Ca^2+^ level increase (Robb et al., 1994) or Gαi/o leads to a cAMP level decrease (Saudou et al., 1990; Robb et al., 1994; Chatwin et al., 2003). OA is more potent to evoke the Gαq-mediated downstream pathway, while TA is more potent to evoke the Gαi/o mediated downstream pathway. The broad diversity of OA receptors (OARs) and their different downstream signaling pathways highlights the intricate nature of OA’s involvement in regulating numerous physiological processes and behaviors. This complexity also complicates efforts to link individual receptor types to specific functions, making a comprehensive analysis of OARs challenging.

**TABLE 1:** Overview of octopamine receptors.

Receptor class	Receptor name	Receptor name abbreviation	Mode of action	CG number
α-adrenergic-like receptors	Octopamine receptor in mushroom bodies	Oamb	Ca^2+^ level increase via Gαq (Han et al., 1998; Balfanz et al., 2005) Induced Ca^2+^ oscillations (Hoff et al., 2011; Balfanz et al., 2005) Ca^2+^/Calmodulin-dependent protein kinase II activity (CaMKII) (Lee et al., 2009)	CG3856
	α2-adrenergic-like octopamine receptor	Octα2R	cAMP levels decrease via Gαi/o (Qi et al., 2017)	CG18208
β-adrenergic-like receptors	Octopamine β1 receptor	Octβ1R	cAMP level increase via Gαs (Maqueira et al., 2005; Balfanz et al., 2005) cAMP levels decrease via Gαi/o (Koon and Budnik, 2012)	CG6919
	Octopamine β2 receptor	Octβ2R	cAMP level increase via Gαs (Maqueira et al., 2005)	CG33976
	Octopamine β3 receptor	Octβ3R	cAMP level increase via Gαs (Maqueira et al., 2005)	CG42244
Tyramine 1 class receptor	Octopamine-Tyramine receptor	Oct-TyrR	Ca^2+^ level increase via Gαq (Robb et al., 1994) (OA more potent than TA) cAMP level decrease via Gαi/o (Saudou et al., 1990; Robb et al., 1994; Chatwin et al., 2003) (TA more potent than OA)	CG7485

List of all octopamine receptors with receptor class, receptor name, abbreviation, mode of action (G-protein downstream signaling upon octopamine binding), and CG number of the gene. OA, octopamine; TA, tyramine.

Previous data on OA and OARs focused mostly on only OA, individual OARs, or incomplete sets of OARs. Here, we compiled a complete collection of transgenic lines to apply the same assays for OA and each OAR. Not only is this the first complete set for all known OARs, but they were also all generated using the same genetic tool—the Trojan Exon developed by Diao et al. (2015). The Trojan Exon lines were generated from the MiMIC collection (*Minos-*mediated integration cassette; Venken et al., 2011) by inserting the Trojan Exon cassette at the MiMIC docking site via recombination-mediated cassette exchange. The Trojan Exon is described as a “coding intron” since it is inserted into an intronic region and contains a splice acceptor and a splice donor, thereby masking it as an exon. Moreover, the cassette encodes a Gal4 for gene-specific targeting, which is translated separately from the endogenous protein. This integration event produces a truncated protein, the length of which is determined by the insertion site within the gene. Such truncation can compromise the functionality of the endogenous protein, while concurrently enabling precise visualization of its native expression pattern via a Gal4-dependent reporter.

With this set of Trojan Exon OA and OAR lines, we observed labeling of each OAR in the central (CNS) and the peripheral nervous system (PNS), and Octβ2R-specific signal in somatic muscles. Bulk RNA-sequencing analysis allowed us to identify additional, previously unknown expression locations, including larval lymph gland, pupal brain, eye, and salivary gland, and adult glia, olfactory projection neurons, and neuronal subsets in the protocerebral bridge. For the first time, we generated an anatomical map of Octα2R in the larva. We observed large overlapping expression patterns of different OARs in various tissues and cell populations throughout the body, from larva to adult. We found that larval hatching ability and larval survival are impaired in *Octβ1R*, *Octβ3R*, and *Oct-TyrR* Trojan lines. Pupal mortality is increased in Trojan *Oct-TyrR*. These results suggest that the involvement of OA during development is greater than previously thought. We compared our results with findings from previous studies and, together with a molecular evaluation of the Trojan Exon OA and OAR lines, provide a complete and validated set of lines for analyzing the OA system beyond this study.

## Materials and methods

### Fly strains

Fly strains were reared on standardized cornmeal medium at 25 °C, 65 % relative humidity, and in a 14:10 h light:dark cycle. The animals were consistently staged by transferring adults regularly to fresh vials, and we controlled the number of parent animals and offspring per vial to minimize variability due to crowding or nutritional differences. Trojan Exon octopamine and octopamine receptor lines were obtained from the BDSC: Oamb (RRID:BDSC_67506), Octα2R (RRID:BDSC_67636), Octβ1R (RRID:BDSC_76677), Octβ2R (RRID:BDSC_67511), Octβ3R (RRID:BDSC_76680), Oct-TyrR (RRID:BDSC_77735) and Tβh (RRID:BDSC_76655). The first three lines carried the TM3, Sb,^1^ Ser^1^ balancer chromosome with no visible marker in larvae; accordingly they were re-balanced over TM6B, *Hu, e*^1^, *Tb*^1^ using the double balancer *w^–^;Bl/CyO, dp*^lvl^*, pr^1^, cn^2^; TM2, Ubx, e*^S^*/TM6B, Hu*, *e*^1^, *Tb*^1^ obtained from the Galizia lab (see also Gonzalez et al., 2016). Control lines were CantonS (WT) and yellow white (y_1_ w*). Only homozygous individuals were used for experiments. Each experiment was performed at 25 °C.

### Oviposition

Freshly hatched or max. 1 day old female flies of each genotype were paired with WT male flies in a 1:1 ratio to ensure a high copulation probability and maximize the yield of laid eggs. After a mating period of 72 h, individual female flies were placed on oviposition plates (Sarstedt, 82.1135.500, Ø 35 mm) filled with 2.5% agarose (w/v) (VWR-Life Science, CAS 9012-36-6), 3% sucrose (w/v) (Roth, CAS 57-50-1) and 30% store-bought apple juice (Sachsen Obst, 100% fruit). The oviposition plates were topped with 2% activated baker’s yeast solution. 24 h later, the number of laid eggs was counted. The sample size is 16 per genotype (one female per n).

### Viability

The oviposition assay was repeated with female and male flies of the same genotype and up to 4 female flies per oviposition plate. The increased number of females ensured a proper number of eggs and later larvae to assess larval and pupal mortality. Female flies were allowed to lay eggs for 24 h, and after another 24 h, the number of hatched larvae was counted. From these plates, 30 hatched larvae were then transferred to standard food vials and monitored closely. The number of pupae and emerging flies was quantified three times a day over a period of 16 days. We did not distinguish between the sexes of the animals, as we are not aware of any sex-specific differences in octopamine and octopamine receptor expression at the larval stage. Hatching rate describes the number of hatched larvae per total number of eggs. Larval mortality is the percentage of larvae that did not enter the pupal stage. Pupal mortality is the percentage of flies that did not hatch from the pupa. Sample size is 16 per genotype and 30 larvae per n, resulting in 480 larvae per genotype, similar to previous studies (Schou, 2013; Lee et al., 2015; Orsted et al., 2019).

### Locomotion assay

The protocol used was modified from Bhatt and Neckameyer (2013). Third-instar foraging-stage larvae were washed with tap water to remove residual food and transferred to a Petri dish (Sarstedt, 82.1472, Ø 92 mm) filled with 2.5% agarose (w/v). After 30 s of acclimatization, single larvae were filmed for 75 s at a rate of 20 fps with an area scan camera (Basler, 106909, lens: 25 mm) and the software Pylon viewer (v 6.3.0; Basler). For a total duration of 1 min, each peristaltic wave was counted for 15 larvae per genotype. This experiment was performed blindly.

### Feeding assay

Feeding behavior was assessed via the rate of mouth hook contractions of third-instar foraging-stage larvae on a Petri dish filled with 2.5% agarose (w/v) and topped with 5 ml of 2% activated baker’s yeast solution. Yeast solution prevented larvae from moving around, ensuring that the recorded mouth hook contractions were a result of feeding behavior. Before the experiment, larvae were washed with tap water to remove residual food. Larvae were allowed to adjust to the new surroundings for 30 s and afterwards were filmed for 75 s with the same setup and software as for the locomotion assay. Each mouth hook contraction over a period of 1 min was counted for 15 individual larvae per genotype. This experiment was performed blindly.

### Olfactory and gustatory preference tests

Experiments were performed as described in Apostolopoulou et al. (2013). For the gustatory preference test, Petri dishes were filled with a thin layer of pure 2.5 % agarose (w/v) on one half and 2.5 % agarose (w/v) mixed with the appetitive stimulus [fructose (Roth, 57-48-7; 2 M) or arabinose (Sigma Aldrich, 10323-20-3; 2 M)] on the other half. The middle of the Petri dish was marked as a 10 mm-wide stripe separating the sugar and pure agarose sides. In this area, 30 larvae were initially placed and were given 5 min of free motion before being counted. Three groups emerged: larvae on the sugar side (#sugar), larvae on the pure agarose side (#agarose), and larvae in the middle. The total number of larvae resulted from the number of larvae in each group (#total). The preference index was calculated as follows: #sugar minus #agarose divided by #total.

Olfactory preference tests were performed on a Petri dish filled with 2.5% agarose (w/v) and one empty custom-made Teflon container (4.5 mm diameter) opposite a second container filled with 10 μl of the odors amyl acetate [Sigma Aldrich, 628-63-7; diluted 1:50 in paraffin oil (Sigma Aldrich, 8012-95-1)] or 1-octanol (Sigma Aldrich, 111-87-5; undiluted). The odor containers were closed with a perforated lid. 30 larvae were transferred to the middle of the Petri dish and were given 5 min of free motion before being counted. Again, three groups emerged: larvae located on the odor side (#odor), larvae on the side with an empty container (#empty container), and larvae in the middle (10 mm wide stripe in the middle of the Petri dish). The total number of larvae resulted from the number of larvae in each group (#total). Preference index is calculated as follows: #odor minus #empty container divided by #total. Positive preference indices represent an attraction toward a gustatory or olfactory stimulus. Sample size is 16 per genotype and 30 larvae per n, resulting in 480 larvae per genotype.

### Anatomical expression analysis

Trojan Exon lines were crossed with *20xUAS-6xmCherry/CyO; nsyb-LexA, LexAop-GFP* to investigate native fluorescence signal, omitting the need for immunolabeling.

First-instar larvae were collected in phosphate-buffered saline (PBS; Sigma Aldrich, P4417) and starved for 1 h. Afterwards, they were placed in 4 % bleach for 10 min, rinsed twice with H_2_O and incubated for another 5 min in H_2_O. Larvae were fixed in 4% paraformaldehyde in PBS (PFA; Sigma Aldrich, F8775) for 15 min at room temperature and 15 min on ice. A washing step included incubation in 3 % Triton-X in PBS (PBT; Sigma Aldrich, X100) for 15 min, repeated three times. Finally, larvae were washed three times in PBS for 15 min. Specimens were embedded in VECTASHIELD^®^ PLUS (Vector Laboratories, H-2000).

Third-instar larvae were collected in PBS and starved for 2 h. Larvae were then heat-fixed by immersion in 70 °C water for 1 min and subsequent cooling in cold water. After drying, samples were mounted in Vectashield and imaged immediately.

Brains of third-instar wandering-stage larvae were dissected in PBS on ice and fixed in 4% PFA for 25 min under vacuum at room temperature. Afterwards, brains were washed six times in 3% PBT and six times in PBS. Brains were mounted on poly-L-lysine (Sigma Aldrich, P1524-25MG) coated coverslips and embedded in Vectashield. Specimens were scanned at a Confocal laser scanning microscope LSM800 by Zeiss with ZEN 2.3 software and processed for publication with ImageJ (National Institutes of Health, Bethesda, Maryland).^1^ Co-labeling was assessed by counting overlapping signal in cell bodies in the calyx and innervated glomeruli of the antennal lobe of the OAR (mCherry) and reference channel (GFP). The mean was calculated from six hemispheres per genotype, and the standard error of the mean (SEM) was calculated as standard deviation divided by the square root of the sample size.

### Gene models for Trojan Exon lines

Gene sequences, transcripts, and annotations for *Tβh* and octopamine receptor genes were obtained via the FlyBase Sequence Downloader (Öztürk-Çolak et al., 2024; release: FB2019_05). Conserved domain predictions were performed with the NCBI Conserved Domain Database (Supplementary Table 1) using the nucleotide sequence of each isoform of the genes. The Trojan Exon Cassette Sequence was acquired from Supplementary Information from Diao et al. (2015). To annotate the sequence of the Trojan Exon, we followed Diao et al. (2015), supplementing this with data from previous publications (Venken et al., 2011; Diao and White, 2012; Diao et al., 2015). The locations of the transgenic insertions can be found in the respective FlyBase entries. Insertion and visualization of the Trojan Exon construct sequence into the receptor gene sequences were done using Benchling (Supplementary Table 1).

### Molecular evaluation—PCR and qualitative RT-PCR

Genomic DNA was isolated using the kit NucleoSpin^®^ Tissue; DNA, RNA, and protein purification; Genomic DNA from Tissue (Macherey-Nagel, 740952.250) following the manufacturer’s protocol. RNA was isolated using TRIzol Reagent (Invitrogen, 15596026) following the manufacturer’s protocol. The second step comprised DNase digestion (Roche, 04 716 728 001) followed by RNA purification using the RNeasy^®^ MinElute^®^ Cleanup kit (Qiagen, 74204), which was performed as stated in the manual. The RevertAid First Strand cDNA Synthesis kit (Thermo Scientific, K1621) was used for cDNA synthesis (performed as in the manufacturer’s protocol).

*Taq* PCR Master Mix (QIAGEN, 201443) was used to amplify target regions according to the manufacturer’s protocol. Primer sequences can be found in Supplementary Table 1. PCR products were separated during gel electrophoresis [1% agarose gel (Sigma Aldrich, A9539), 1xTBE buffer, MassRuler DNA Ladder Mix (Thermo Scientific, SM0403)]. Gel extraction of amplified products was performed with QIAquick Gel Extraction Kit (QIAGEN, 28706) and was sequenced by Microsynth Seqlab^®^. Online tools and websites used are listed in Supplementary Table 1.

### Quantitative real-time RT-PCR

Total RNA from third-instar larvae was isolated using QIAzol (Qiagen, 79306) followed by DNA digestion using RNase-Free DNase Set (Qiagen, 79256) and purification using RNeasy Mini Kit (Qiagen, 74104); all steps were performed according to the manufacturer’s protocol. 250 ng RNA was reverse transcribed using SuperScript™ III First-Strand Synthesis SuperMix for qRT-PCR (Invitrogen, 11752050). Gene expression was measured by quantitative real-time RT-PCR and the TaqMan methodology (Dm02139710_g1 for *Octβ2R* and Dm01825538_m1 for *Oct-TyrR*). Fluorescence was detected on an ABI PRISM 7500 sequence detector (Applied Biosystems, Foster City, CA, USA) according to the manufacturer’s instructions (Applied Biosystems). In short, we used the standard detection method. The conditions were as follows: 50.0 °C for 2 min, 95.0 °C for 10 min, and 40 cycles of 95.0 °C for 15 s and 60.0 °C for 1 min. Data were collected at the 60.0 °C step in each of the 40 cycles. The total reaction volume was 10 μl, and a standard curve was used on each plate. mRNA expression was calculated relative to the mRNA expression of *Rpl32* (Applied Biosystems; assay Dm02151827_g1 for *Rpl32*) and to y^1^w* control larvae.

### Bulk RNA-seq analysis

Raw sequencing reads were obtained from the European Nucleotide Archive.^2^ The RNA-seq runs used in this study are listed in Supplementary Table 2. Quality control of raw FASTQ files was performed using FastQC (v0.11.9), and reads were pseudo-aligned to the reference transcriptome (Drosophila melanogaster, genome assembly BDGP6.54, Ensembl release 113) using Salmon (v1.10.2) in quasi-mapping mode with sequence-specific and GC bias correction enabled (–seqBias, –gcBias). Transcript-level expression estimates were imported into R (v4.3.3) using the importIsoformExpression function from the IsoformSwitchAnalyzeR package (v2.2.0). Gene-level expression values were derived by summing transcript-level TPMs for each gene. Genes with low expression were filtered before downstream analyses. Specifically, genes were retained only if they showed a gene-level expression of ≥ 5 TPM in at least 3 samples. For comparative and visualization purposes, gene expression values were summarized as median TPM per tissue and developmental stage. TPM values were log-transformed where indicated for improved visualization. Gene expression levels were compared between individual tissues and all remaining tissues within the same developmental stage. Expression differences are reported as log2 fold change (log2FC), where positive values indicate higher expression in the focal tissue. Statistical significance was assessed using non-parametric Wilcoxon tests. *P*-values were adjusted for multiple testing using the Benjamini–Hochberg procedure, and false discovery rate (FDR) values are reported (Supplementary Table 3). FDR < 0.05 was considered statistically significant.

### Statistical analysis

**FIGURE 2:**
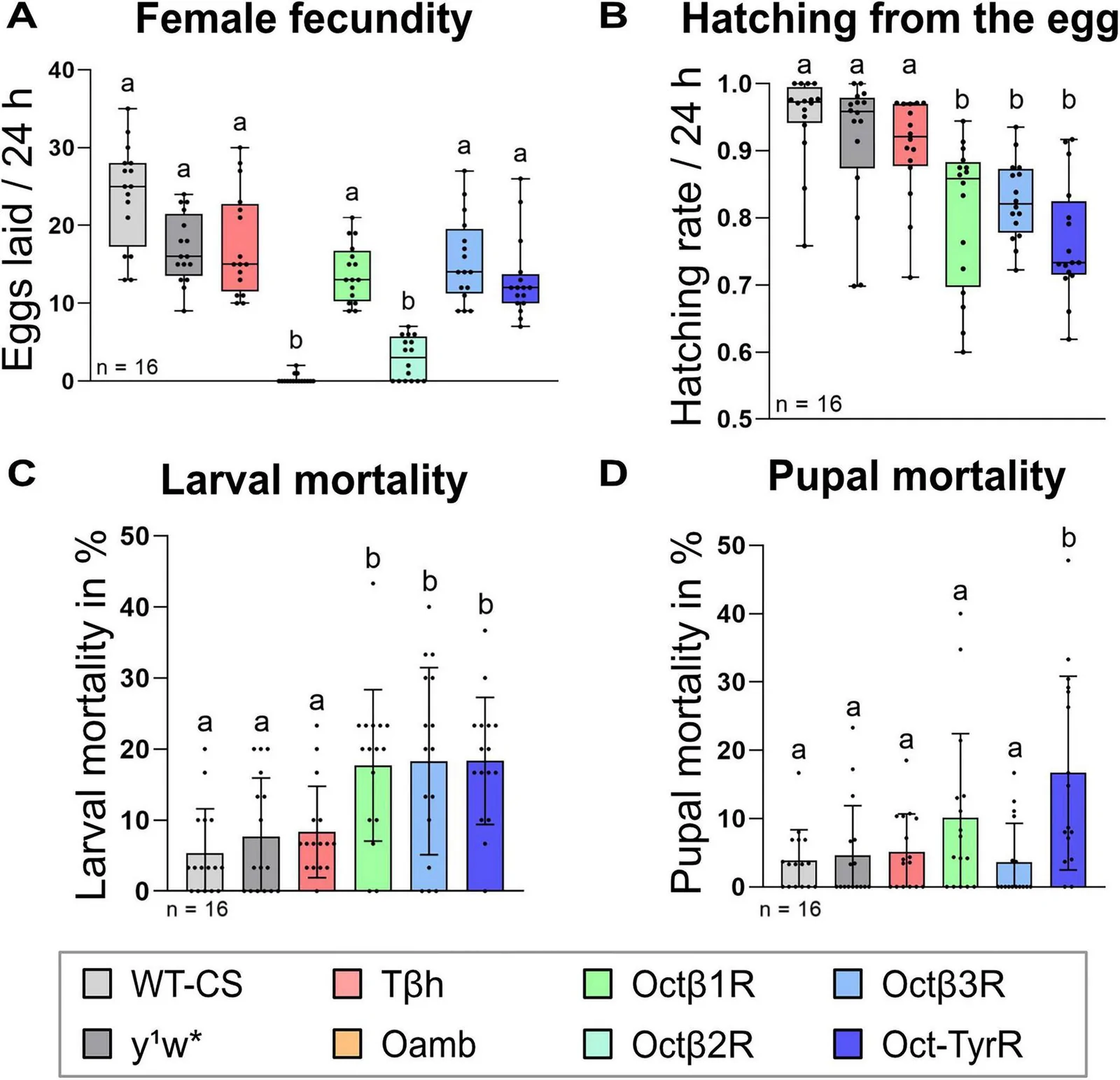
Trojan Exon octopamine receptor lines exhibit reduced overall survival. **(A)** Female fecundity was assessed by allowing mated 3–4-day-old single female flies to lay eggs for 24 h. Any defects in male reproductive capacity were ruled out by allowing females to mate with WT males. The number of laid eggs was diminished only in Trojan *Oamb* (*p* < 0.0001) and *Octβ2R* (*p* < 0.0001) females compared to the genetic control y_1_w*. **(B)** In a second approach using homozygous animals, the ability of larvae to hatch from their eggs was analyzed. Females laid eggs for 24 h, then were removed from the assay plate. After another 24 h, the number of hatched and unhatched eggs was counted. The hatching rate was calculated as the number of hatched larvae per the total number of laid eggs. The number of hatched larvae of Trojan *Octβ1R* (*p* = 0.0081), *Octβ3R* (*p* = 0.0158), and *Oct-TyrR* (*p* = 0.0002) was significantly reduced. Trojan *Oamb* and *Octβ2R* had to be excluded because homozygous females did not lay enough eggs for comparison with the control. Trojan *Octa2R* was excluded since all animals were heterozygous. Hatched first-instar larvae were used to determine larval and pupal mortality in **(C,D)**. **(C)** Larval survival was assessed by transferring 30 first-instar larvae to a standard food vial and counting the number of emerging pupae. Larval mortality was calculated as the number of larvae that did not pupate (in %). Trojan *Octβ1R* (*p* = 0.0246), *Octβ3R* (*p* = 0.0388), and *Oct-TyrR* (*p* = 0.0121) lines showed increased larval mortality. **(D)** Pupal survival was based on the number of flies that were able to hatch from the pupa. Pupal mortality was calculated as the number of adult flies that did not hatch (in %). Trojan *Oct-TyrR* (*p* = 0.0069) showed increased pupal mortality. For **(A–D)**, data were analyzed with a Kruskal-Wallis test and Dunn’s multiple pairwise comparison. Experimental groups were compared to the genetic background line, y_1_w*. Letters are used to represent statistical differences between groups, where the same letter indicates no significant difference and different letters indicate significant differences.

**FIGURE 3:**
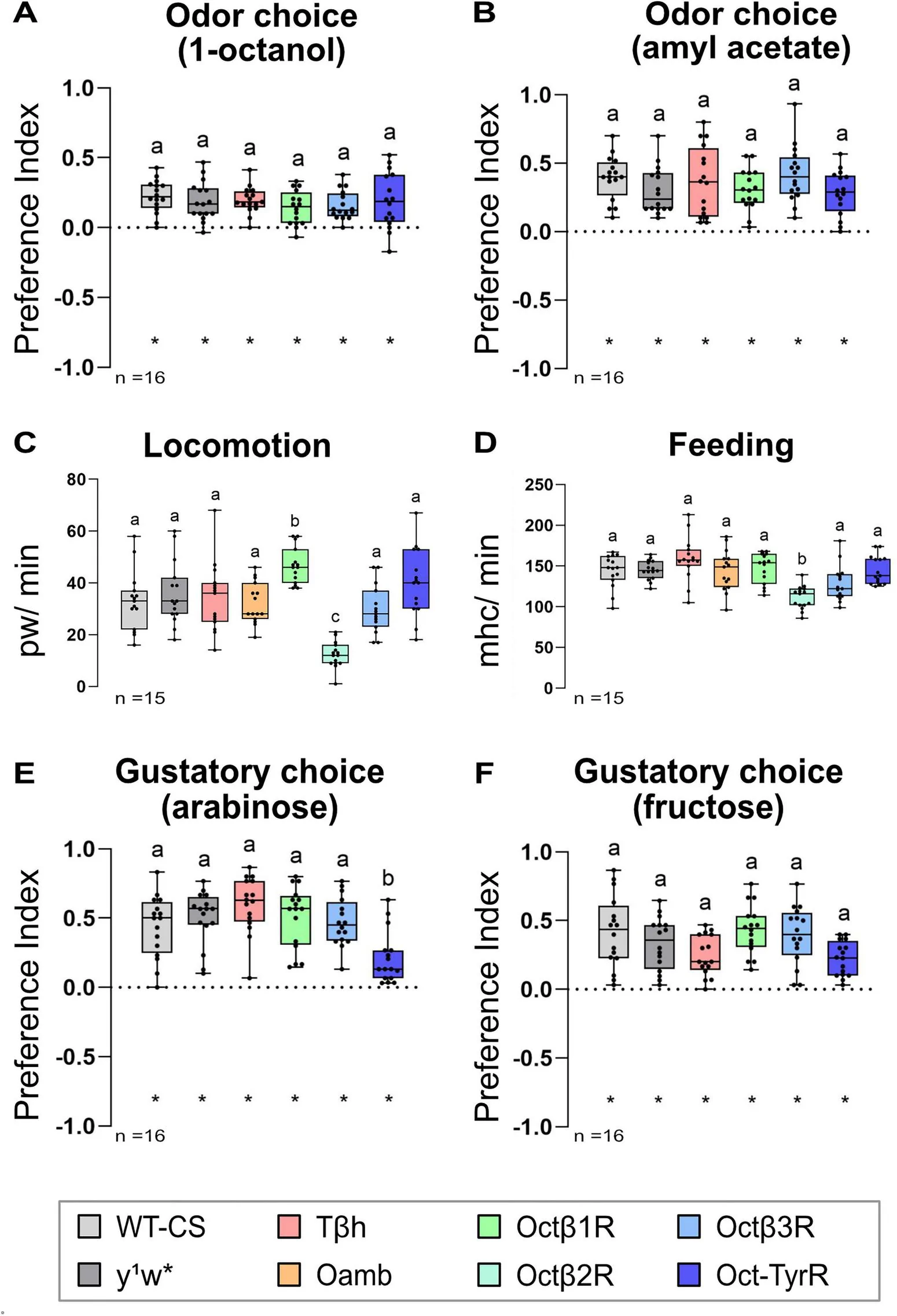
Locomotion is impaired in Trojan *Octβ1R* and *Octβ2R*. Odor and taste preference tests were conducted using 30 larvae per data point, for which one preference score was calculated. To determine locomotion and feeding rate, one larva was used per data point. **(A)** In a choice assay, larvae’s preference for the odor 1-octanol (undiluted) over an empty container after 5 min of exposure is shown. Each Trojan Exon octopamine receptor line showed a preference for the odor similar to that of the control. **(B)** In a choice assay, larvae’s preference for the odor amyl acetate (1:50 dilution in paraffin oil) over an empty container after 5 min of exposure is shown. Each Trojan Exon octopamine receptor line showed a preference for the odor similar to that of the control. **(C)** Locomotion behavior was analyzed by quantifying the peristaltic waves per minute (pw/min) of a moving larva on an agarose plate without any food source. Trojan *Octβ1R* showed significantly more pw/min (*p* = 0.0257), while *Octβ2R* showed significantly less pw/min (*p* < 0.0001). **(D)** Mouth hook retractions can be utilized as an indirect readout of feeding. Surrounding the larva with a rich food environment makes it possible to distinguish between mouth hook retractions used for crawling and for feeding. Eliminating the need to move forward, in this assay, larval mouth hook retractions were used to determine the ability of food uptake. Trojan Exon octopamine receptor lines did not differ from the control in their feeding rate. Only Trojan *Octβ2R* showed a significantly decreased number of mouth hook retractions per minute (mhc/min; *p* = 0.0008). **(E)** The preference for the sugar arabinose (2 M) over neutral agarose after 5 min of food uptake was assessed. Arabinose has a sweet taste but is not nutritious and has no caloric value for the larva. Only Trojan *Oct-TyrR* showed a reduced preference (*p* = 0.0036) yet preferred this sugar over plain agarose. (F) The preference for the sugar fructose (2 M) over neutral agarose after 5 min of food uptake was assessed. Fructose is a sweet and nutritious sugar. For **(A–C, F)**, data were analyzed with a one-way ANOVA followed by a Tukey honestly significant difference post hoc test. For **(D,E)**, data were analyzed with a Kruskal-Wallis test and Dunn’s multiple pairwise comparison. For **(A,B,E,F)**, the Wilcoxon signed-rank test was applied to compare groups against the chance level. For each panel, experimental groups were compared to the genetic background line, y_1_ w*. Letters are used to represent statistical differences between groups, where the same letter indicates no difference and different letters indicate significant differences.

GraphPad Prism 8.0 (GraphPad Software Inc.) was used for statistical analysis and visualization. First, the normal distribution of data points was assessed with the Shapiro-Wilk test. If a normal distribution was assumed, a one-way ANOVA followed by a Tukey honestly significant difference post hoc test for pairwise comparison between more than two groups was applied. If assumptions of normal distribution were not met, data were analyzed with a Kruskal-Wallis test and Dunn’s multiple pairwise comparison for more than two groups. Experimental groups were compared to the genetic background line, y* w*. The Wilcoxon signed-rank test was chosen to compare groups against the chance level. Significance level was set at 0.05. Box plots are shown with 25 and 75% quantiles as box boundaries, while whiskers display the complete data set (min to max values). The median is indicated as the middle line, and each dot represents a data point. Bar plots show the mean and standard deviation of the data set. Each dot represents a data point. The following statistical tests were applied: Figure 2: For A-D, data were analyzed with a Kruskal-Wallis test and Dunn’s multiple pairwise comparison. Figure 3: For A, B, C, and F, data were analyzed with a one-way ANOVA followed by a Tukey honestly significant difference *post-hoc* test. For D and E, data were analyzed with a Kruskal-Wallis test and Dunn’s multiple pairwise comparison. For A, B, E, and F, the Wilcoxon signed-rank test was applied to compare groups against the chance level.

## Results

**FIGURE 4:**
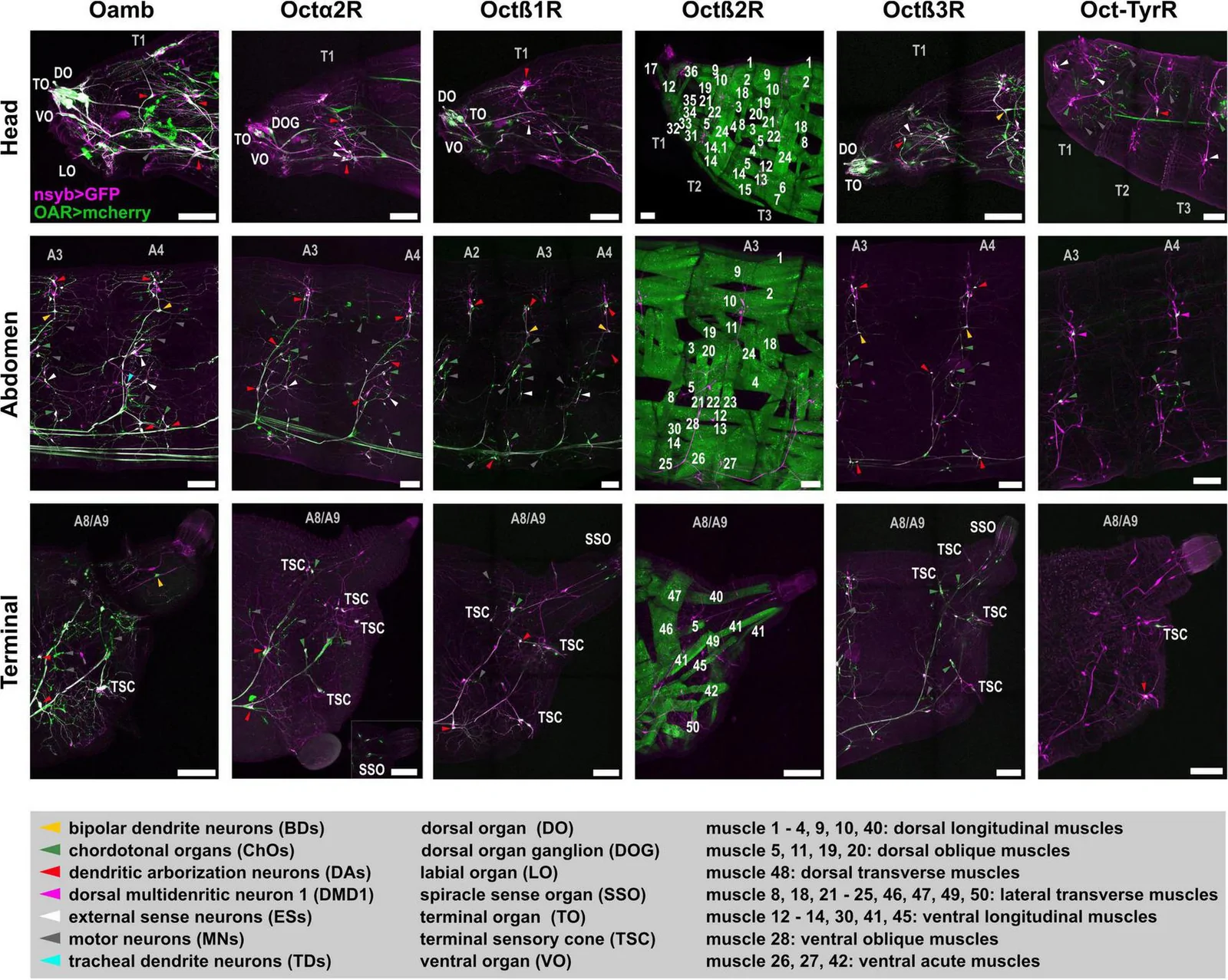
Expression pattern of each octopamine receptor throughout the body of Trojan Exon lines. Using native fluorescence, the expression pattern of each octopamine receptor (green via mCherry) and neuronal Synaptobrevin, as a reference channel (magenta via GFP), is shown. For each Trojan Exon line, the head (pseudocephalon: PCE; and up to thoracic segment 3: T3), abdominal segment 3 (and A4), and abdominal segment A8/9 are shown. In Trojan *Octα2R*, the signal in SSO is shown in an extra inset due to technical reasons. In Trojan *Octβ2R*, muscle labeling was observed. Additional muscle labeling of *Octβ2R* from the ventral and dorsal views of the larva, as well as signal in the terminal organ, is shown in Supplementary Figure 1. Expression pattern in whole first-instar larvae is shown in Supplementary Figure 2 for each receptor, including an overview of the expression patterns (Supplementary Figure 3). Scale bar: 100 μm.

**FIGURE 5:**
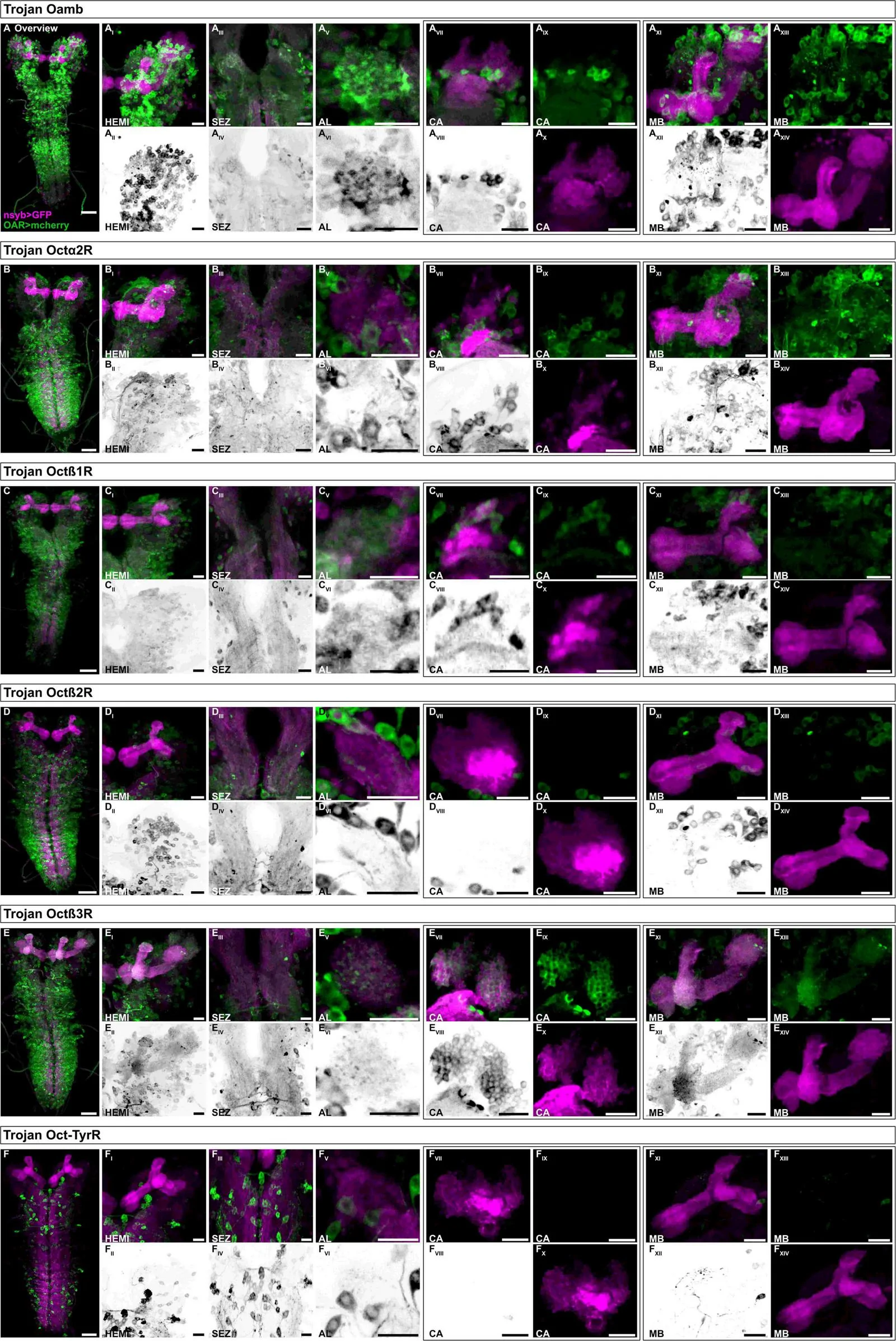
Expression pattern of each octopamine receptor in the central nervous system (CNS) of Trojan Exon lines: **(A)** *Oamb*, **(B)** *Octα2R*; **(C)** *Octβ1R*; **(D)** *Octβ2R*; **(E)** *Octβ3R*; **(F)** *Oct-TyrR*. Using native fluorescence, the expression patterns of each octopamine receptor (green via mCherry) and neuronal Synaptobrevin, as a reference channel (magenta via GFP), are shown. For all lines, the first panel shows an overview of the CNS. Close-ups of one hemisphere (HEMI), the subesophageal zone (SEZ), the antennal lobe (AL), the calyx (CA), and the mushroom body (MB) are shown from left to right. Below each panel is a grayscale image of the mCherry channel only to show the innervation patterns without the reference channel (panels II, IV, VI, VIII, and XII). For the CA and MB, a panel with only mCherry (IX and XIII) and GFP (X and XIV) channels is shown. Scale bar: 50 μm (Overview), 20 μm (HEMI, SEZ, AL, CA, MB panels).

**FIGURE 6:**
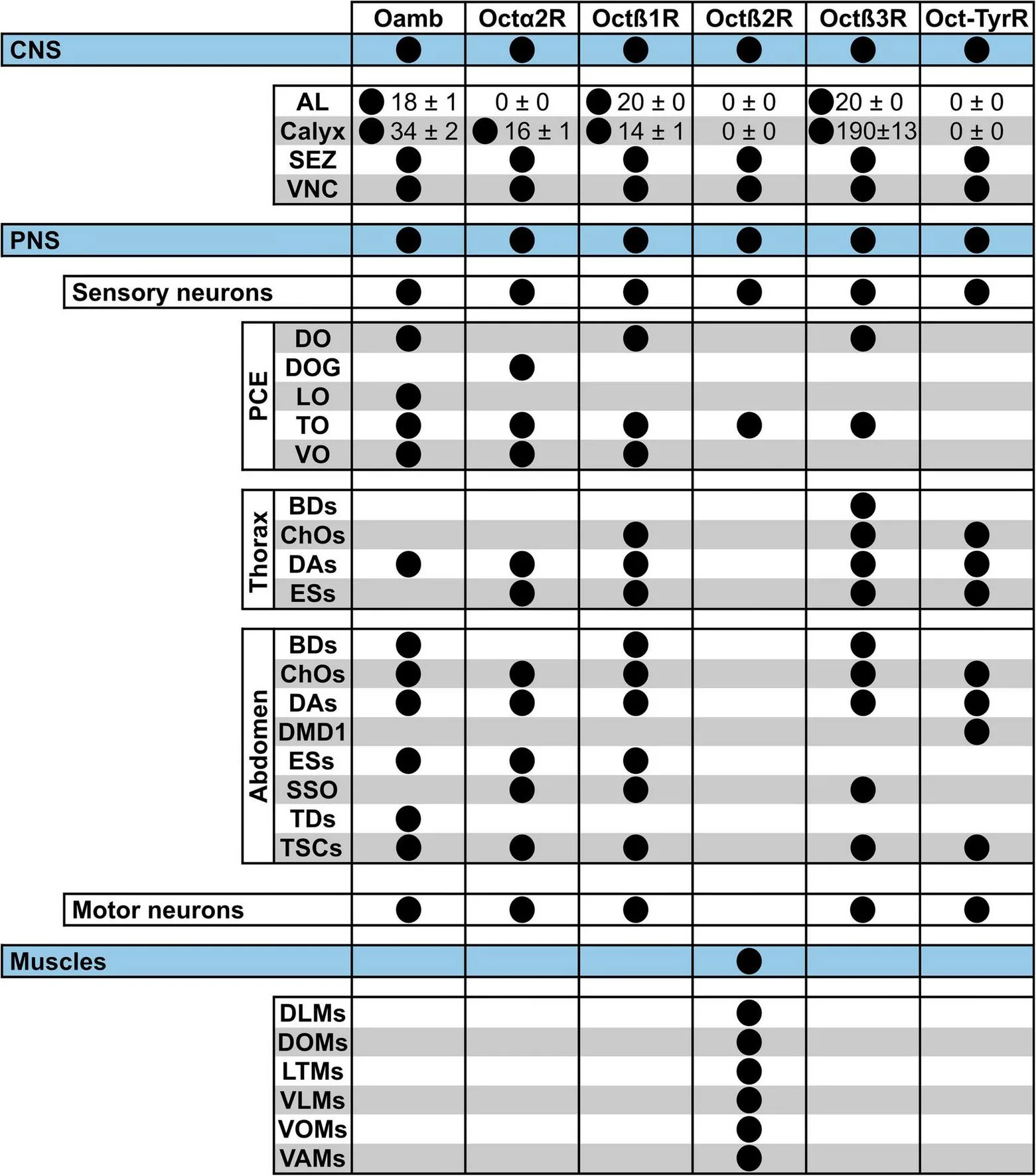
Overview of expression patterns of Trojan Exon octopamine receptor lines throughout the body. Summary of expression in the central nervous system (CNS), peripheral nervous system (PNS, split into sensory neurons and motor neurons), and somatic muscles of the body wall of each Trojan Exon octopamine receptor line. Numbers indicate co-labeling of OARs with glomeruli of antennal lobe, and cell bodies of Kenyon cells in the calyx of the mushroom body (mean number ± SEM; n = six hemispheres of three brains). AL, antennal lobe; BDs, bipolar dendritic neuron; ChOs, chordotonal organs; DAs, dendritic arborization neurons; DLM, dorsal longitudinal muscle; DMD1, dorsal multidendritic neuron 1; DO, dorsal organ; DOG, dorsal organ ganglion; DOM, dorsal oblique muscle; ESs, external sensory neuron; LO, labial organ; LTM, lateral transverse muscle; PCE, pseudocephalon; SEZ, Subesophageal zone; SSO, spiracle sense organ; TDs, tracheal dendrite neuron; TO, terminal organ; TSCs, terminal sensory cones; VAM, ventral acute muscle; VLM, ventral longitudinal muscle; VNC, Ventral nerve cord; VO, ventral organ; VOM, ventral oblique muscle.

**FIGURE 7:**
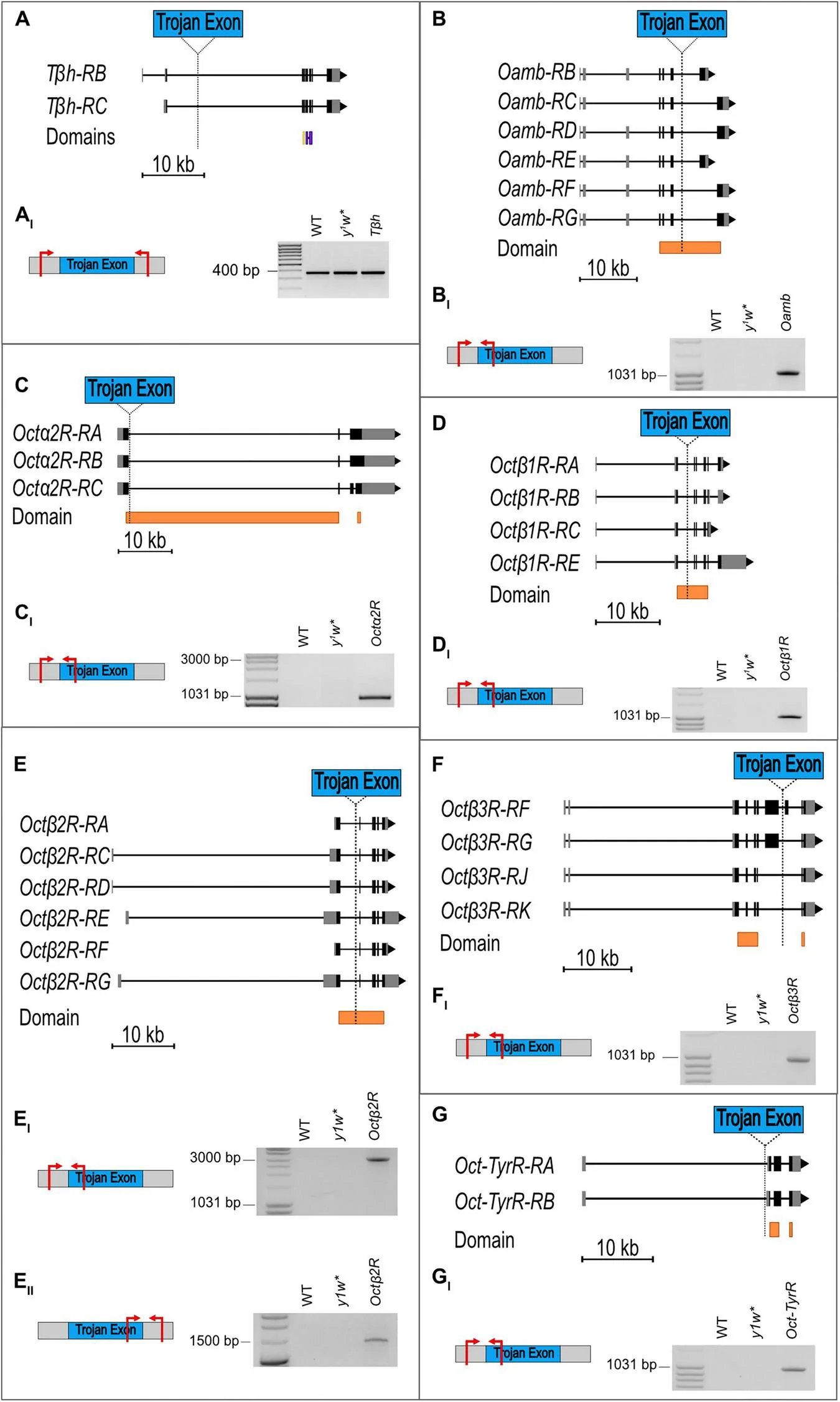
Evaluation of the correct insertion of the Trojan Exon. **(A–G)** A schematic of the genes *Tβh, Oamb, Octα2R, Octβ1R, Octβ2R, Octβ3R* and *Oct-TyrR*. For each gene, the structure of each isoform with 5′ UTR (gray), Exons (black), Introns (space between Exons), and 3′ UTR (gray) is depicted. At the bottom, the predicted conserved domains and their location in the gene are marked. The insertion location of the Trojan Exon is indicated in the isoforms (dashed line). **(A_I_–G_I_)** On the left, a schematic shows the location of the primer (red arrows) used for PCR corresponding to the Trojan Exon (blue) in the gene (gray). On the right, visualization of the amplified product during PCR. For **(A_I_)**, first, a PCR with one primer in the gene and one in the Trojan Exon (same strategy as for the octopamine receptors) showed no amplified products (data not shown). In a second approach, a product of the same size was observed in the WT, y^1^w* and Trojan *Tβh*. Sequencing revealed that the three amplicons were identical (Supplementary Figure 4). For **(B_I_–G_I_,E_II_)**, no product was amplified in the WT and y^1^w* control since the Trojan Exon, in which one primer is binding, is not present. For each Trojan Exon line, a product was obtained and sequenced, which confirmed the insert to be present and at the correct location (Supplementary Figures 5–12). Only for Trojan *Octβ2R*, the amplicon was of a bigger size than expected (around 3000 bp; **E_I_**). **(E_II_)** Due to that, a second PCR was conducted to check for the 3′ side of the Trojan Exon. Again, additional sequences were found (around 130 bp). Sequencing confirmed the insertion of additional sequences at the 5′ **(E)** and 3′ **(E_II_**) side of the Trojan Exon (Supplementary Figures 10–12), although in a BLAST analysis, those sequences were not found to be coding (Supplementary Table 9, 10).

**FIGURE 8:**
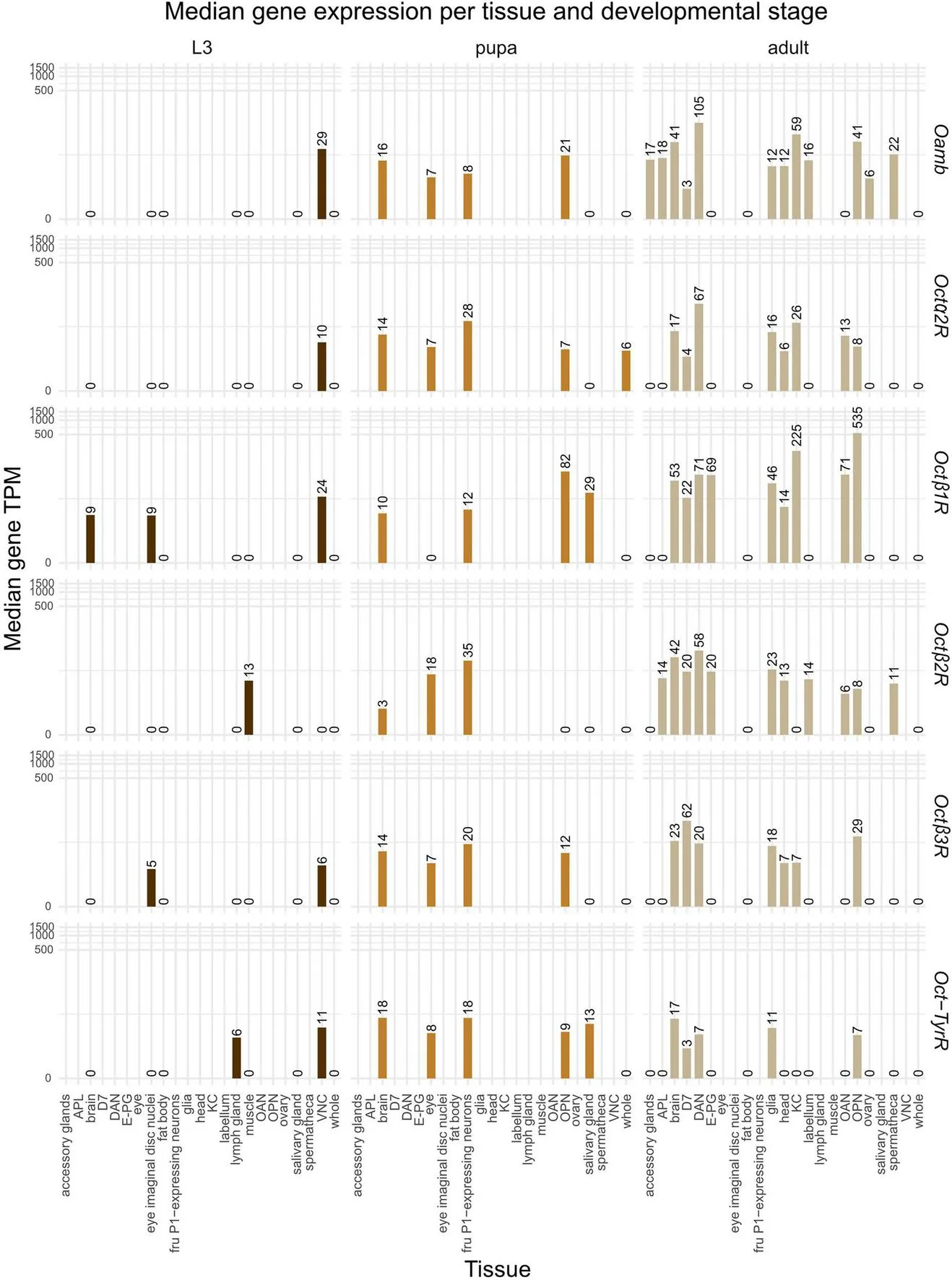
Median gene expression across tissues and developmental stages. Median transcript abundance (TPM) was calculated for all analyzed genes in each tissue and developmental stage (third-instar larva (L3), pupa including prepupa, and adult). Tissue–stage combinations with no data are left blank. Median TPM values were log-transformed for visualization. Sample numbers per tissue and stage are as follows: third-instar larva: brain (hemispheres and VNC; *n* = 56), eye imaginal disc nuclei (*n* = 3), fat body (*n* = 35), lymph gland (*n* = 3), muscle (*n* = 14), salivary gland (*n* = 17), ventral nerve cord (VNC; *n* = 8), whole body (*n* = 183). Pupa (including prepupa): brain (*n* = 6), eye (*n* = 18), fru P1-expressing neurons (*n* = 16; only males), olfactory projection neurons (OPN; *n* = 3), salivary gland (*n* = 13), whole body (*n* = 76). Adult: olfactory projection neurons (OPN; *n* = 3), anterior paired lateral neuron (APL; *n* = 5), brain (*n* = 74), Δ7 neurons (D7; *n* = 4), dopaminergic neurons (DAN; *n* = 14), E-PG neurons (*n* = 4), fat body (*n* = 12), glia (*n* = 7), head (*n* = 201), Kenyon cells (KC; *n* = 9), labellum (*n* = 3), octopaminergic neurons (OAN; *n* = 154), ovary (*n* = 81), spermatheca (*n* = 6), whole body (*n* = 444).

In order to use the Trojan Exon OA and OAR lines for the analysis of the OA system, we investigated whether known phenotypes could be reproduced. We tested for I) the prominent role of Tβh, Oamb, and Octβ2R in female sterility (Monastirioti et al., 1996; Lee et al., 2003; Monastirioti, 2003; Lim et al., 2014; Rezaval et al., 2014; Deady and Sun, 2015; Li et al., 2015), II) the role of *Octβ3R* in pupation formation (Ohhara et al., 2015) and III) disrupted larval locomotion in *Tβh, Octβ1R*, *Octβ2R*, and *Oct-TyrR* larvae (Saraswati et al., 2004; Fox et al., 2006; Koon and Budnik, 2012; Selcho et al., 2012). We integrated these tests in the (A) developmental assays, comprising female fecundity, larval hatching, as well as larval and pupal survival (Figure 2); (B) behavioral assays, comprising chemosensation, locomotion, and feeding behavior (Figure 3); and (C) anatomical mapping, comprising the central and peripheral nervous system as well as non-neuronal tissue (Figures 4–6). A molecular analysis supported the evaluation of the Trojan Exon OA and OAR lines (Figure 7). In addition to the developmental, behavioral, and anatomical experiments with OA and OAR lines, transcript profiling based on bulk RNA-sequencing data revealed previously unknown expression locations and demonstrated quantitative differences in expression across distinct developmental stages, tissues, and cell types (Figure 8).

### Octopamine receptors contribute to the animal’s viability—from egg to adult

First, we performed a general assessment of the animal’s viability over the different developmental stages. Embryonic mortality was examined based on the ability of the first-instar larva to hatch from the egg. Larval and pupal mortality were used to evaluate survival rates. Additionally, female sterility was analyzed by applying an egg-laying test, in which the number of eggs laid by individual flies within 24 h was counted. To exclude potential effects arising from male reproductive system defects (as has been reported for Oamb; Zhou et al., 2012; Luo et al., 2014), Trojan OAR females were mated with WT males. A significantly reduced number of eggs was observed for Trojan *Oamb* and *Octβ2R* compared to the genetic control. In contrast, no reduction was seen for Trojan *Tβh*, *Octβ1R*, *Octβ3R*, and *Oct-TyrR* (Figure 2A). Due to the limited number of experimental animals resulting from the small number of eggs laid (Figure 2A) and the low percentage of homozygous animals (Supplementary Table 4), further analysis of larval development in Trojan *Oamb, Octα2R*, and *Octβ2R* was not possible. For all other groups, we assessed the ability of larvae to hatch from the egg and calculated a hatching rate, which is defined as the number of hatched larvae divided by the total number of eggs. This time, females were mated with males of the same genotype to analyze only homozygous larvae. The hatching rate was found to be reduced in Trojan *Octβ1R*, *Octβ3R*, and *Oct-TyrR* compared to control animals, while no reduction was observed in *Tβh* (Figure 2B). Subsequently, the survival of larvae and pupae was monitored. Larval mortality was assessed as the percentage of larvae that did not enter the pupal stage. A significant increase was observed in Trojan *Octβ1R*, *Octβ3R*, and *Oct-TyrR* (Figure 2C). Pupal mortality, which indicates the percentage of animals unable to hatch from the pupa, showed that only Trojan *Oct-TyrR* had a reduced number of hatched flies, reflected in an increased pupal mortality (Figure 2D). Raw data and statistical analysis for all data presented in Figure 2 can be found in Supplementary Table 5. So, on the one hand, the Trojan *Oamb* and *Octβ2R* lines reproduced the well-known female sterility phenotype; on the other hand, for the first time, it was shown that Octβ1R and Oct-TyrR are involved in larval hatching and larval survival. Even the survival of pupae was impaired in Trojan *Oct-TyrR*. We also observed a decreased survival rate of larvae in Trojan *Octβ3R*, which could be related to the reported impairment of pupation (Ohhara et al., 2015). Nevertheless, for the first time, we showed that Octβ3R is involved in larval hatching.

### Analysis of larval foraging behavior

In addition to physiological mechanisms, changes in behavior can also explain the decreased viability of Trojan OAR lines observed in Figure 2. Therefore, we aimed to analyze the role of the OARs in one such behavior—foraging. During the larval stage, energy requirements are high due to a body mass increase of about 200 times (Bakker, 1959). Consequently, foraging plays an essential role in the life of larvae, ensuring their survival and progression into metamorphosis (Bakker, 1961; Ohnishi, 1979). In this study, we subdivide the larval foraging behavior into (I) the perception of olfactory cues of a potential food source (Figures 3A,B), (II) locomotion on pure agarose (Figure 3C), (III) the ability of food uptake (Figure 3D), and (IV) the gustatory preference for food sources that are generally indicative of high energy supply (Figures 3E,F). Olfactory cues can provide a first indication of a potential food source and help the larva to navigate in their environment and initiate and/or maintain contact with a substrate (reviewed in Cobb, 1999; shown in Gomez-Marin et al., 2011; Gershow et al., 2012). Here, we tested the preference for the two odors, amyl acetate and 1-octanol, which can be associated with a food source in an olfactory associative learning and memory assay (reviewed in Gerber and Stocker, 2007; shown in Selcho et al., 2009; Pauls et al., 2010; Schleyer et al., 2011; Apostolopoulou et al., 2013; Weber et al., 2023a; Weber et al., 2023b). We used the same experimental setup and odor concentrations as in Apostolopoulou et al. (2013). Larvae from each Trojan Exon line showed a preference for the two odors, similar to control animals (Figures 3A,B). It is important to note that due to the small number of laid eggs, which resulted in a limited number of experimental animals, Trojan *Oamb* and *Octβ2R* could not be included in these mass assay experiments.

After perceiving an appetitive odor cue, the crawling behavior is directed more specifically toward a potential food source (Fishilevich et al., 2005; Louis et al., 2008). In a locomotion assay, we evaluated the crawling ability by quantifying the peristaltic waves (pw) as a read-out of rhythmic motor patterns (Gjorgjieva et al., 2013; Liu et al., 2023). Tested animals were neither exposed to external stimuli nor treated in any other way. While Trojan *Octβ1R* exhibited an increased number of pw/min, Trojan *Octβ2R* showed significantly less pw/min than the control animals (Figure 3C). Given that these assays analyze individual larvae, it was possible to include the relatively small number of surviving animals of Trojan *Oamb* and *Octβ2R*.

The next assay focused on assessing the feeding behavior of the larvae. In contrast to crawling behavior, feeding primarily relies on contractions of the cephalopharyngeal muscles, which are separate from the somatic segment muscles in the abdomen (Schoofs et al., 2010). However, mouth hook contractions (mhc) are also required for crawling behavior. In the feeding assay, larvae were placed in a nutrient-rich environment that eliminated the need to search for food. Mouth hook contractions serve as a straightforward indicator of feeding behavior (Bhatt and Neckameyer, 2013). Most Trojan Exon lines showed no change in feeding rate; only *Octβ2R* larvae had a significantly reduced number of mhc per minute compared to control animals (Figure 3D).

Gustatory preferences can indicate whether a larva is able to identify beneficial sources of nutrition, such as sweet and high-calorie food (Rohwedder et al., 2012). In a choice assay, larvae were presented with sugar on one side and tasteless, non-nutritious agarose on the other side. We used two different sugars: (I) fructose, which is sweet and has a high caloric value, while (II) arabinose is only sweet but non-nutritious. All Trojan Exon lines preferred the sugars over the neutral agarose (Figures 3E,F). However, Trojan *Oct-TyrR* showed a significantly reduced preference for arabinose compared to the control. Please note that due to the small number of laid eggs, which resulted in a limited number of experimental animals, Trojan *Oamb* and *Octβ2R* were not included in these mass assay experiments. Raw data and statistical analysis for all data in Figure 3 can be found in Supplementary Table 6.

### Expression pattern of Trojan Exon OAR lines in the larva

Utilizing the simultaneously driven Gal4 expression in the Trojan Exon lines, we anatomically mapped the expression of each OAR in neuronal and non-neuronal cells. Two protocols were applied, one to evaluate the respective expression throughout the body (Figure 4) and the other in the CNS (Figure 5) of third-instar larvae. For each receptor, the head (pseudocephalon and up to thoracic segment 3), abdominal segment 3 and/or 4, and abdominal segment A8/9 are shown (Figure 4). We map only unambiguous signals, mostly at the body surface, but the labeling of deep-seated tissues cannot be excluded. The first panel in Figure 5 shows an overview of the CNS. Close-ups of one hemisphere (HEMI), the subesophageal zone (SEZ), the antennal lobe (AL), the calyx (CA), and the mushroom body (MB) are shown from left to right. Below each panel is a grayscale image only of the channel showing receptor expression. For the CA and MB, additional panels show receptor expression and reference signal separated from each other. To evaluate co-labeling of OARs with the CA and the AL, we counted the number of cell bodies of intrinsic Kenyon Cells (KC) in the CA and glomeruli of the AL with OAR signal, utilizing six hemispheres per genotype (Supplementary Table 7).

Trojan *Oamb* showed labeling in the dorsal organ (DO), labial organ (LO), terminal organ (TO), and ventral organ (VO) in the pseudocephalon (PCE) (Figure 4). Each segment, from thoracic segment 1 (T1) to abdominal segment 8/9 (A8/9), featured signal in dendritic arborization neurons (DAs) as well as motor neurons (MNs). Additional labeling was found in bipolar dendrite neurons (BDs), chordotonal organs (ChOs), external sense neurons (ESs), and tracheal dendrite neurons (TDs) in the abdomen, as well as in the terminal sensory cones (TSC) in A8/9. Trojan *Oamb* was observed to be the only OAR expressed in TDs. The CNS showed labeling from the hemispheres (Figures 5AI,II) to the subesophageal zone (Figures 5AIII,IV). The larval AL is organized in 21 glomeruli (Ramaekers et al., 2005). Approximately 18 ± 1 glomeruli of the AL showed labeling (Figures 5AV,VI). The MB consists of approximately 600–650 intrinsic KC per hemisphere, whose axons form the lobes of the MB (Ito and Hotta, 1992; Ramaekers et al., 2005). About 34 ± 2 KC cell bodies in the CA showed double-labeling (Figures 5AVII–XIV).

In Trojan *Octα2R*, both TO and VO labeling can be found in the PCE (Figure 4). While the DO itself is not labeled, signals were detected in a group of sensory neurons belonging to the dorso-lateral group of the TO, which have their cell bodies within the associated ganglion (DOG). DAs, ESs, and MNs showed Trojan *Octα2R* expression in both thoracic and abdominal segments. ChOs were solely labeled in the abdomen. In terminal abdominal segments A8/A9, TSC, and spiracle sense organ (SSO) were labeled. Multiple cells in the CNS showed a signal. Only a few KC (approximately 16 ± 1), likely born embryonic (Pauls et al., 2010), were found to express Octα2R (Figures 5BVII–XIV).

Trojan *Octβ1R* showed expression in DO, TO, and VO in the PCE and in ChOs, DAs, ESs, and MNs in each thoracic and abdominal segment (Figure 4). Additionally, BDs were labeled in the abdominal segments, and TSCs and SSO were observed to be labeled. In the CNS, we found many cell bodies labeled, from the hemispheres to the VNC. About 20 ± 0 glomeruli of the AL and approx. 14 ± 1 KC cell bodies showed Trojan *Octβ1R* signal (Figures 5C,CI–XIV,CV).

Trojan *Octβ2R* signals can be found in somatic muscles of the body wall across each thoracic and abdominal segment (Figure 4). In the PCE, two intersegmental muscles showed labeling: muscle 12, which runs from dorsal T1 to ventral PCE, and muscle 17, which extends from laterofrontal PCE to dorsal T2. In the prothorax (T1), we identified dorsal longitudinal muscle 36 and ventral longitudinal muscles 31–35, which are associated with the mouthparts (Hooper, 1986). In the mesothorax (T2), signal was observed in dorsal longitudinal (muscle 1–4, 9, 10), dorsal oblique (muscle 5 and 19; muscle 20 is probably covered by muscle 19), lateral transverse (muscle 8, 18, 21, 22, 24; muscle 23 is probably covered by muscle 22 and 24) and ventral longitudinal muscle (muscle 14). Only the dorsal oblique muscle 30 appears to be not labeled. In the metathorax (T3), we identified dorsal longitudinal (muscle 1–4, 9, 10), dorsal oblique (muscle 5, 19, 20), lateral transverse (muscle 8, 18, 21, 22, 24; muscle 23 is probably covered by muscle 22 and 24), ventral longitudinal (muscle 6, 7, 12, 13, 14) and ventral oblique muscles (muscle 15). Only ventral acute muscle 26 and dorsal oblique muscle 30 showed no signal. Each muscle of the body-wall in abdominal segments A1-7 showed expression in the Trojan line and can be summarized as follows since the muscle pattern is identical for each segment: dorsal longitudinal (muscle 1–4, 9, 10), dorsal oblique (muscle 5, 11, 19, 20), lateral transverse (muscle 8, 18, 21–25), ventral longitudinal (muscle 6, 7, 12–14, 30), ventral oblique (muscle 15–17, 28) and ventral acute (muscle 26, 27, 29). Due to their position, the labeling of muscles 6, 7, 15, 16, 17, and 29 is shown in Supplementary Figure 1A. Muscle 25 is the only muscle not present in A1 (Zarin et al., 2019) and hence was not found during the expression analysis. In segment A8/9, dorsal longitudinal muscle 40, dorsal transverse muscle 48 (Supplementary Figure 1B), lateral transverse muscles 46, 47, 49, and 50, ventral longitudinal muscles 41 and 45, as well as ventral acute muscle 42, were identified. These muscles can be grouped into I) associated with the terminal spiracle (muscles 40, 41, and 42) and II) associated with the anal division (muscles 45, 46, 47, 49, and 50), depending on their position (Wipfler et al., 2013). The ring-shaped muscle 43, which surrounds the terminal spiracles, showed no labeling. According to Wipfler et al. (2013), we found a muscle labeled as number 5, although it was not described in detail. Due to the uncertainty of their position, the identity of two additional muscles (44 and 51) was not determined. Muscles 44 and 51 have been previously described but not visualized (Wipfler et al., 2013). Muscle identity was determined based on the work of Wipfler et al. (2013) for PCE and A8/9, Hooper (1986) for T1-3, and Zarin et al. (2019) for A1-7. Additionally, we identified a signal in at least one sensory neuron in the TO (Supplementary Figure 1C). While additional expression may also occur in other sensory neurons, motor neurons, or non-neuronal cells, the strong signal in the somatic muscles could be overshadowing these other signals. The CNS of Trojan *Octβ2R* showed signal in the VNC and the hemispheres (Figures 5D,DI,II). We found single cell bodies around the AL but no innervation (Figures 5DV,VI), and a cell cluster lateral to the vertical lobe of the MB, but no labeled KC (Figures 5DXI–XIV).

In Trojan *Octβ3R*, signals were observed in both the DO and TO within the PCE (Figure 4). Expression was detected in the thoracic and abdominal segments in certain neuron types, including BDs, ChOs, DAs, ESs (only thorax), and MNs. In segment A8/9, ChOs and MNs, as well as SSO and TSCs, were labeled. In the CNS, the expression signal encompassed all segmental ganglia within the VNC and the hemispheres (Figures 5E,EI,II). The SEZ showed cell bodies along the midline and laterally toward the VNC (Figures 5EIII,IV). While we observed innervation of the AL (about 20 ± 0 glomeruli; Figures 5EV,VI), many KCs in the CA and the lobes of the MB were distinctively labeled (approx. 190 ± 13; Figures 5EVII–XIV). Additional cell bodies in the hemispheres, around the MB, expressed Trojan Octβ3R (Figures 5EI,II,XI–XIV).

In thoracic and abdominal segments, we observed ChOs, DAs, ESs, and MNs in Trojan *Oct-TyrR* larvae (Figure 4). Signal in the dorsal multidendritic neuron 1 (DMD1) was observed in the abdomen, and other OARs were not expressed in this particular neuron type. Similar to most OARs, Oct-TyrR showed expression in TSCs in segment A8/9. The CNS showed the most specific expression pattern among the Trojan Exon OAR lines. Cell bodies in the abdominal segments were positioned laterally, while the thoracic segments had additional cell bodies along the midline (Figure 5F). In the SEZ, we found cell bodies specifically near the esophagus (Figures 5FIII,IV). The hemispheres showed only little Oct-TyrR signal and no distinct labeling of the AL or MB (Figures 5FI,II,V–XIV).

A summary of the expression locations throughout the entire body and the CNS can be found in Figure 6. Additional whole-body scans of first-instar larvae show similar expression patterns compared to the third-instar larvae (Supplementary Figures 2A–F, including a summary in Supplementary Figure 3). For ChOs, TSCs, and somatic muscles, the same cellular composition could be found. For DAs, DOs, ESs, MNs, SSO, and TOs, the first-instar larval organizational pattern is maintained in third-instar larvae, though supplemented by additional Trojan OAR lines (DAs: Octβ1R, Oct-TyrR; DO: Octβ1R; ESs: Oct-TyrR; MNs: Octα2R, Octβ1R, Oct-TyrR; SSO: Octβ1R; TOs: Octβ2R). It remains unclear whether these differences represent developmental changes in OAR expression or technical limitations.

### Molecular analysis reveals the absence of Trojan Exon in the Tβh line

*Tβh* lines are known to exhibit severe egg laying impairments as well as larval locomotor deficits (Monastirioti, 2003; Saraswati et al., 2004; Selcho et al., 2012), prominent behavioral phenotypes that were not observed in Trojan *Tβh*. Moreover, during expression pattern analysis, no signal was observed in the brain, which contradicted an earlier study that found labeling throughout the CNS, applying immunohistochemistry (Selcho et al., 2012). In Trojan *Tβh*, the Trojan Exon is located in the first half of the *Tβh* gene locus (based on FlyBase entry ID FBti0195387), resulting in a premature truncation of the protein (Figure 7A). The conserved domains DOMON (dopamine beta-monooxygenase N-terminal; yellow) and the Copper type II ascorbate-dependent monooxygenase (blue) were predicted to be located downstream of the Trojan Exon insertion site (Supplementary Table 8) and are crucial for the enzymatic function of Tβh (Figure 7A). It can therefore be assumed that the truncated Trojan *Tβh* protein is non-functional, which is why we expected to find previously known phenotypes of *Tβh* lines. Thus, we performed targeted PCR to analyze the Trojan Exon on the genomic level and found that the Trojan *Tβh* line lost the cassette. According to the published insertion site of the Trojan Exon in the *Tβh* gene locus, MI04010, we used a primer pair that surrounded the *Tβh* MI04010 docking site. In the case of the presence of the Trojan Exon, we expected an amplified product of around 4 kb. But, as shown in Figure 7AI, the size of the amplicon was similar to that of the WT and the y^1^w* controls. Sequencing of the amplified PCR product revealed no differences between Trojan *Tβh* and the WT *Tβh* gene locus of the reference genome acquired from FlyBase (Supplementary Figure 4). We repeated the targeted PCR with a different copy of the Trojan *Tβh* fly line and received identical results (data not shown).

Based on these results, we continued analyzing the Trojan Exon OAR lines to ensure the correct insertion of the Trojan Exon. This time, a different primer strategy was applied: based on the MiMIC docking sites (*Oamb*: MI12417, *Octα2R*: MI10227, *Octβ1R*: MI05807, *Octβ2R:* MI13416, *Octβ3R*: MI06217, *Oct-TyrR*: MI03485), one primer was located in the genomic region and one in the Trojan Exon (see schematic in Figure 7 for each OAR). Due to the size of the amplified products, sequencing results, and comparison with the reference genome acquired from FlyBase, we conclude that the location of the Trojan Exon in the *Oamb*, *Octα2R*, *Octβ1R*, *Octβ2R, Octβ3R* and *Oct-Tyr* receptor lines is identical with the corresponding MiMIC docking sites (Figures 7BI,CI,DI,EI,FI,GI; and Supplementary Figures 5–12). Only for Trojan *Octβ2R*, the amplicon size was larger than expected (expected: 1072 bp, observed: ∼ 3,000 bp; Figure 7EI). A second PCR at the 3′ end of the Trojan Exon in the *Octβ2R* gene locus showed a similar result (expected: 1366 bp, observed: ∼1500 bp; Figure 7EII). Sequencing revealed that the additional parts were not endogenous to the *Octβ2R* gene, and BLAST analysis showed no distinct origin (Supplementary Figures 10–12 and Supplementary Table 9, 10). We further analyzed if the additional sequences upstream of the 5′ end of the Trojan Exon were transcribed and could affect the formation of the Trojan Exon. A schematic (not to scale) shows the unknown additional parts (∼ 2,000 bp) right upstream of the Trojan Exon at the genomic level (Supplementary Figure 13A). In a qualitative RT-PCR, primers were designed to bind in the upstream Exon 7 and in the Trojan Exon. If the inserted sequences were excluded from transcription, an amplicon of 532 bp was expected. The observed PCR product, visualized below the schematic, migrated between 500 and 600 bp. Subsequent sequencing of this fragment confirmed the absence of the additional sequences in the *Octβ2R* mRNA of the Trojan *Octβ2R* line (Supplementary Figure 14). Moreover, quantitative real-time RT-PCR was performed to analyze *Octβ2R* mRNA levels. We applied the TaqMan methodology with a probe binding downstream of the inserted Trojan Exon (Supplementary Figure 13A). Only a fraction (∼6%) of *Octβ2R* mRNA was detected compared to the control, showing the decreased expression of this OAR in the Trojan Exon line (Supplementary Figure 13C). Therefore, although additional sequences are present in the genomic locus, our data suggest that they are spliced out and are unlikely to cause off-target effects at the transcript level.

In each tested line, the Trojan Exon is inserted into the first half of the respective OAR gene (Figure 7), suggesting that the Trojan Exon-induced protein truncation leads to the loss of major structural parts of the receptor. Conserved domain prediction analysis revealed that the conserved transmembrane domains of the GPCRs are affected (Figure 7 and Supplementary Table 8), suggesting that the OARs are non-functional and thus that the Trojan Exon OAR lines are suitable for investigating the OAR system. Only for *Oct-TyrR*, the insert location of the Trojan Exon is in an intron in the non-coding 5′ UTR. Since the Trojan Exon must be inserted into an intron between two coding exons, the intended effect of truncating the Oct-TyrR protein and thereby disrupting its functionality is questionable. But quantitative real-time RT-PCR showed that no *Oct-TyrR* transcript was detectable in the Trojan Exon line, indicating that the Oct-TyrR protein is absent (Supplementary Figures 13B,C). Further, due to the insertion location in an intron in the 5′ UTR, the expression pattern obtained during anatomical mapping may not correspond to the endogenous pattern of Oct-TyrR.

Overall, insertion sites were confirmed in most Trojan Exon OARs, making these lines suitable genetic tools for interfering with the OA system. Even though the insertion site of the Trojan Exon in *Octβ2R* is compromised, the line seems to be valid based on our mRNA analysis. For Trojan *Oct-TyrR*, we found that the targeted protein disruption takes place even if it may not have been in the intended manner. But due to the insertion location in an intron in the 5′ UTR, Gal4-induced expression may not correspond to the endogenous Oct-TyrR pattern. Only Trojan *Tβh* cannot be used for further studies because it lacks the Trojan Exon.

### Gene expression data

All results obtained so far are based on experiments with Trojan Exon lines. In a different approach, we utilized bulk RNA-sequencing data retrieved from the European Nucleotide Archive to focus on OAR expression location and quantity in third-instar larvae, pupae, and adults, each in different tissues and cell populations (Figure 8 and Supplementary Table 3).

In third-instar larva, expression of Oamb (log2FC = 2.29, FDR < 0.0001), Octα2R (log2FC = 3.40, FDR < 0.0001), Octβ3R (log2FC = 2.88, FDR < 0.001), and Oct-TyrR (log2FC = 3.55, FDR < 0.0001) was more pronounced in the VNC than the hemispheres, in contrast to Octβ1R (log2FC = 0.00, FDR = 0.53) that was detected throughout the brain (hemispheres and VNC). Increased levels of Octβ1R (log2FC = 3.31, FDR = 0.09) and Octβ3R (log2FC = 2.63, FDR = 0.02) were observed in eye imaginal disc nuclei, while Octβ2R (log2FC = 0.63, FDR = 0.006) showed increased expression in third-instar larva muscle compared to the other OARs and other cells and tissues. A yet unknown expression location was found for Oct-TyrR (log2FC = 2.85, FDR < 0.0001) in the lymph gland.

In the pupa, every OAR was detected in the brain and in a neuronal subset, the fru P1-expressing neurons of the male. The pupal eye showed expression of each OAR except Octβ1R, and olfactory projection neurons (OPN) were found to express all OARs except Octβ2R at increased levels. In the salivary glands of the prepupa, only Octβ1R (log2FC = 4.90, FDR < 0.0001) and Oct-TyrR (log2FC = 1.08, FDR = 0.05) were observed. Throughout the body, Octα2R was the most abundant OAR.

In the adult, whole head samples showed expression of each OAR except Oct-TyrR (log2FC = 0.00, FDR = 0.62). But in the brain, Oct-TyrR (log2FC = 4.19, FDR < 0.0001) transcript levels were increased as well, indicating that head tissue surrounding the brain does not express Oct-TyrR as much as the other OARs. In some neuron populations, every OAR was observed at high abundance, namely: Δ7 neurons (D7) of the protocerebral bridge, dopaminergic neurons (DAN), glia, and olfactory projection neurons (OPN). Other neuron populations were found to express some OARs more than others, such as KC (Oamb, Octα2R, Octβ1R and Octβ3R); octopaminergic neurons (OAN, Octα2R, Octβ1R and Octβ2R); the anterior paired lateral neuron (APL; Oamb and Octβ2R) and the so-called E-PG neurons in the central complex (Octβ1R and Octβ2R). Similarly, non-neuronal tissue was found to express max. two OARs at elevated levels: labellum and spermatheca express the same OARs (Oamb and Octβ2R), and the accessory gland and ovary express only Oamb. In addition, due to the assignment of cell identity in RNA-sequencing data, we were able to find so far unknown expression locations in neuronal cells: APL (Oamb, Octβ2R), DAN (Octα2R, Octβ1R, Octβ2R, Octβ3R, and Oct-TyrR), Δ7 neurons (Oamb, Octα2R, Octβ1R, Octβ2R, Octβ3R and Oct-TyrR), E-PG neurons (Octβ1R and Octβ2R), glia (Oamb, Octα2R, Octβ1R, Octβ3R, and Oct-TyrR), KC (Octα2R), OPN (Oamb, Octα2R, Octβ1R, Octβ2R, Octβ3R, and Oct-TyrR), and in non-neuronal cells: labellum (Oamb, Octβ2R).

Interestingly, Octβ1R was found at exceptionally high transcript levels in adult OPN (median gene TPM: 535, log2FC = 7.4, FDR = 0.013) and in KC (median gene TPM: 225, log2FC = 6.1, FDR < 0.0001) compared to all other expression levels in the different tissues and cells, as well as for other OARs. Only Oamb in DAN (median gene TPM: 105, log2FC = 4.7, FDR < 0.0001) showed a similar high transcript level.

Overall, each OAR was expressed in fewer tissues and cells in third-instar larva compared to the pupa and adult. And although the median transcript abundance in general was low in the whole body in each stage (with Octα2R being the only exception in the pupa), expression was elevated in specific tissues and cell populations, varying from third-instar larva to adult.

## Discussion

The Trojan Exon enables analysis of a functionally disrupted target gene and localizes its gene product. Using this genetic tool, we were able to break down the analysis from the entire OA circuit to the receiver side, focusing on each receptor individually. We identified novel phenotypes of OARs in larval hatching and the survival of larvae and pupae. Figure 1 summarizes the published and novel functions of the OARs across the developmental stages. Moreover, we expanded the expression map of OARs to (I) cells and tissues that were not considered to be a potential place of action of OA, (II) to the first-instar larva, and (III) to Octα2R, which was not reported before. Bulk RNA-seq analysis allowed the identification of additional putative sites of expression across developmental stages, such as Oct-TyrR in larval lymph gland and adult glia. Although transcript abundance does not permit direct conclusions regarding protein concentration, quantitative mRNA analysis provides an alternative approach for estimating gene expression levels in cases where antibodies are unavailable. Supplementary Table 11 provides an overview of the expression patterns in the third-instar larva from this study combined with previous findings. The molecular evaluation of the Trojan Exon OARs provides a solid basis for future investigations of the OAR system. Likewise, the results show that Trojan Exon lines generally require molecular validation to ensure that the gene of interest is disrupted as intended.

### Molecular considerations regarding the OAR Trojan Exon lines

Prominent phenotypes of *Tβh* lines are female sterility (Monastirioti et al., 1996; Monastirioti, 2003; Rezaval et al., 2014) and impaired larval locomotion (Saraswati et al., 2004; Fox et al., 2006; Selcho et al., 2012), among others. We analyzed the performance of Trojan *Tβh* for these two selected traits and couldn’t find any impairments (Figures 2A, 3C). An anatomical analysis of Tβh in the Trojan *Tβh* line following crosses with several UAS reporters revealed no signal (data not shown). Molecular evaluation revealed that the Trojan *Tβh* line had lost the Trojan Exon (Figure 7AI). Reordering the line showed the same results. Therefore, the Trojan *Tβh* line is not appropriate for studying the function of OA in *Drosophila*.

Molecular evaluation of the Trojan *Octβ2R* line revealed additional sequences 5′ and 3′ of the Trojan Exon (Figures 7EI,II). However, we conclude that despite these genetic inconsistencies, the Trojan *Octβ2R* is a suitable tool to investigate this receptor. The additional sequences are not present at the mRNA level (Supplementary Figure 14), and the Trojan Exon leads to decreased *Octβ2R* transcript levels (Supplementary Figure 13C). Residual *Octβ2R* expression of approximately 6% may reflect low-level alternative splicing that bypasses the Trojan Exon. The utility of the Trojan *Octβ2R* line is supported both at the behavioral and anatomical levels. Behaviorally, it recapitulated previously reported phenotypes, including female fecundity and impaired larval locomotion (Figures 2A, 3C; Lim et al., 2014; Li et al., 2015; Koon and Budnik, 2012). Anatomically, Trojan *Octβ2R* expression showed larval muscle labeling as reported before (Figure 4; El-Kholy et al., 2015).

For the Trojan *Oct-TyrR*, no mRNA was detected (Supplementary Figure 13C). The absence of *Oct-TyrR* mRNA may therefore account for the observed developmental and behavioral phenotypes (Figures 2B–D, 3E), much like the previously reported chemotaxis defect (Ma et al., 2016). But due to the insertion of the Trojan Exon in the *Oct-TyrR* 5′ UTR (Figure 7G), the expression pattern shown in Figures 4, 5F might not resemble the endogenous one. Thus, the Trojan *Oct-TyrR* should be used with caution, despite showing CNS and MNs expression patterns comparable to those previously reported (Stowers, 2011).

### Oamb

Given the established role of Oamb in the female reproductive system (Lee et al., 2003; Deady and Sun, 2015), the Trojan Oamb line’s egg-laying phenotype (Figure 2A), combined with the molecular validation (Figure 7), confirms its suitability as a tool for further studies, thereby substantiating our expression analysis data (Figures 4, 5A). We observed labeling throughout the larval CNS with Oamb being more abundant in the VNC than the hemispheres (Figure 8). The primary olfactory processing center, the AL, and the learning and memory center, the MB, were labeled, suggesting potential function in these specified structures (Figures 5A, 6). The PNS showed signal from the head to the tail: beginning in the PCE, gustatory (TO, VO), olfactory (DO), mechanosensory (DO, LO, TO, VO), thermosensory (DO), chemosensory (oxygen; TO), and osmosensory (TO) organs were identified (for further information on the function of the different organs, see Rist and Thum, 2017; Richter et al., 2023). Thoracic and abdominal segments were found to have signal in neurons and organs for proprioception (DAs; Ferreira Castro et al., 2020; He et al., 2019; ChOs; Caldwell et al., 2003), innocuous touch (DAs; Hu et al., 2017; Tsubouchi et al., 2012), as well as noxious stimuli such as cold, heat, touch, light, or acidity (DAs; Tsubouchi et al., 2012). Recently, a function for Oamb in DAs was found in nociceptive plasticity (Boivin et al., 2026). ESs in the abdomen indicate further involvement in the perception of mechanical and/or chemical stimuli (Jan and Jan, 1993). Labeled TDs that wrap tracheal branches and TSCs at A8/9 suggest a role in the respiratory system since these structures are associated with the perception of CO_2_ and/or oxygen (Bodmer and Jan, 1987; Hückesfeld et al., 2021; Lu et al., 2024; Richter et al., 2023). In the neuromuscular system, BD neurons associated with somatic muscles (Bodmer and Jan, 1987) and motor neurons were labeled (also reported by Jetti et al., 2023; Bakshinska et al., 2025). Among the Trojan OAR lines, the Trojan *Oamb* is the most broadly distributed OAR in the larval periphery, suggesting an as- yet unknown function in the sensory system (for further discussion, see the last paragraph).

### Octα2R

The Trojan *Octα2R* line allowed us to generate the first anatomical expression map in the larva (Figures 4, 5B, 6). Interestingly, a small number of KC was labeled, indicating a potential function in learning and memory (approx. 16 ± 1; Figures 5B, 6). In the PCE, Octα2R was observed in the DOG, TO, and VO, suggesting involvement in gustatory, mechanosensory, chemosensory, and/or osmosensory processes. Across the body, we found signals in DAs, ESs, and MNs, as well as in ChOs in the abdominal segments; thus, Octα2R may be associated with proprioception and/or nociception, mechano-/chemosensory, and the neuromuscular system. At the terminus, the TSCs and SSO were labeled. Richter et al. (2023) discussed the function of the TSCs and SSO in the positioning of the larva in a food medium to hide from predators and daylight, but in the same way, avoid suffocation from digging too deep (Kim et al., 2017). Although the exact role of OA in this survival mechanism remains to be elucidated, the labeling of the Trojan *Octα2R* line may serve as an initial indicator of its potential involvement.

### Octβ1R

The Trojan *Octβ1R* line exhibited increased larval mortality (Figure 2C). However, the larval feeding rate remained unaltered (Figure 3D), making it unlikely that insufficient nutrient intake is the primary driver of mortality. Instead, the hyperactivity phenotype (Figure 3C) points toward a fatal energetic mismatch. Since increased locomotion in *Drosophila* significantly raises metabolic rate and accelerates the depletion of energy reserves (Sujkowski et al., 2017), the larvae likely succumb to metabolic exhaustion. Under these conditions, a normal feeding rate appears insufficient to compensate for the high metabolic costs of chronic hyperactivity, ultimately leading to the observed mortality. Beyond these metabolic constraints, the Trojan line labeled various cells in the third-instar larva, suggesting potential functional involvements that may contribute to survival. This labeling was observed across diverse sensory structures, including gustatory (TO, VO), olfactory (DO), mechanosensory (DO, TO, VO), thermosensory (DO), chemosensory (TO), and osmosensory (TO) organs (Figure 4). Larval survival could be affected by a potential function in proprioception (ChOs and DAs), nociception (DAs), mechano-/chemosensation (ESs), or the neuromuscular system (BDs and MNs). Defects within the TSCs and SSO might have resulted in the detrimental positioning of larvae within the food medium, subsequently leading to a misjudgment of oxygen availability. Nevertheless, the labeling in the CNS, particularly in specialized structures such as the AL and MB, remains a crucial factor when analyzing the role of Octβ1R in larval mortality (Figures 5C–CXIV). Specifically, the high abundance of this receptor throughout the CNS and in eye imaginal disc nuclei (compared to other OARs) suggests a functional role in the nervous system and during development (Figure 8). Additionally, earlier studies reported this receptor in the Malpighian tubules, the tracheal system (El-Kholy et al., 2015), the salivary glands (Ohhara et al., 2012), and the eye-antennal discs (Ohhara et al., 2012; Koon and Budnik, 2012). Finally, a potential involvement in the viability of first- and second-instar larvae, or during molting, cannot be excluded.

Bulk RNA-seq analysis revealed that most OARs are expressed at low levels across different tissues and developmental stages. In contrast, Octβ1R exhibited exceptionally high abundance, restricted to specific cell populations: KC and OPN in adults. While Octβ1R in the MB drives aversive learning, its localization in OPN plays a major role in appetitive learning (Sabandal et al., 2020). The exceptional abundance of Octβ1R stands out against the otherwise low expression background in different cells and developmental stages, as well as the other OARs, pointing to its specialized importance in KC and OPN.

### Octβ2R

In the Trojan *Octβ2R* line, we observed labeling of all 29 somatic muscles of abdominal segment 1 and all 30 somatic muscles of abdominal segments 2–7 in the body wall (Figure 4). Since crawling requires the activation of all somatic body wall muscles (Zarin et al., 2019), this widespread labeling pattern accounts for the locomotion deficit of the Trojan Octβ2R line (Figure 3C). Moreover, head muscles (PCE and T1–3) exhibited labeling and are engaged in larval movements like turning or burrowing. In terms of feeding behavior, mouthpart-associated muscles in the prothorax were labeled (Figure 4; Hooper, 1986), and consistently, the Trojan Exon line displayed a reduced feeding rate (Figure 3D). Labeling of the somatic muscles flanking the terminal spiracles and anal division implies an involvement in respiratory and excretory functions (Figure 4). Although Octβ2R has been described in MNs (Koon et al., 2011; Koon and Budnik, 2012; Jetti et al., 2023), no MN labeling was observed in the Trojan Exon line. This discrepancy might arise because delicate neuronal structures were masked by the strong muscular signal. This rationale could also explain the absence of signal in the fat body, intestine, imaginal discs, Malpighian tubules, midgut, salivary glands, and tracheal system, despite expression being detected in earlier studies (Ohhara et al., 2012; El-Kholy et al., 2015). Aside from the muscles, the TO exhibited the only peripheral signal, presumably due to the absence of overlapping muscle fluorescence.

### Octβ3R

In third-instar larvae, Octβ3R labeling was observed in both internal and external sensory organs, responding primarily to chemo- and mechanosensory signals. This indicates that Octβ3R plays a functional role in various sensory modalities, including gustation (TO), olfaction (DO), mechanosensation (DO, ESs, TO), thermosensation (DO), chemosensation (ESs, TO), osmosensation (TO), and proprioception (ChOs, DAs). Additionally, it may be involved in peripheral neurons related to nociception (DAs) and muscle activity (BDs, MNs) (Figure 4). Trojan *Octβ3R* showed labeling in TSCs and SSO, which are believed to respond to changes in oxygen levels or to give mechanosensory feedback when the larvae submerge into the substrate. When this complex information is not properly processed, the larva’s ability to adjust to environmental conditions may be compromised, potentially impacting survival (Figure 2C). Additionally, the CNS expression pattern (Figures 5E, 6) and elevated transcript levels (Figure 8) warrant attention, as does the high abundance in the nuclei of eye imaginal discs (Figure 8). However, as summarized in Supplementary Table 11, previous studies have detected Octβ3R in additional tissues, including various glands, the Malpighian tubules, and the midgut.

During our assessment of larval mortality, we focused on counting the number of emerging pupae rather than quantifying deceased larvae. Previously, Octβ3R was reported to play a crucial role in inducing metamorphosis by regulating ecdysone biosynthesis in the prothoracic gland (Ohhara et al., 2015). However, the pupariation defect observed in our Trojan Octβ3R line was less pronounced than in that earlier report. This discrepancy may be attributed to technical differences or variations in the genetic tools employed; specifically, Ohhara et al. (2015) used phm-22-Gal4 to drive two independent RNAi lines targeting Octβ3R in the prothoracic gland, an approach whose strong pupariation phenotype might be overestimated. Alternatively, because the Trojan Exon is inserted further downstream in the gene, it could potentially allow for the production of a truncated but partially functional receptor. However, this same line provides a distinct learning phenotype that we recently described in detail (Franke et al., 2026), demonstrating its utility for larval olfactory learning experiments. Consequently, further investigations remain necessary to determine the precise temporal and spatial window of Octβ3R function affecting larval survival and pupariation.

### Oct-TyrR

The Trojan *Oct-TyrR* line exhibited reduced survival rates during larval and pupal development (Figures 2C,D). The labeling in ChOs, DAs, DMD1, ESs, and TSCs suggests a potential involvement in proprioception (ChOs, DAs, and DMD1; Heckscher et al., 2015), as well as mechano- and chemosensation and oxygen perception (ESs and TSCs, Figure 4). These sensory roles align with the observed reduction in sugar preference (Figure 3E), a previously reported chemotaxis phenotype, and the altered startle response in *honoka* larvae, a different *Oct-TyrR* line (Ma et al., 2016). Moreover, labeling was observed throughout the body, including the CNS, dorsal aorta, lymph gland, Malpighian tubule, posterior spiracles, salivary gland, and the tracheal system (Stowers, 2011; El-Kholy et al., 2015; Figures 5F, 8). Whether a functional role in the sensory system, the different tissues, or both accounts for the increased larval mortality remains unclear. However, a critical function during early larval stages or during molting cannot be ruled out. Future experiments utilizing stage-specific lethality assays or tissue-specific knockdown will be necessary to delineate the distinct developmental functions of OARs.

Further, we identified a potential role for Oct-TyrR in pupal survival, based on the increased pupal mortality observed in Trojan *Oct-TyrR* (Figure 2D). Interestingly, bulk RNA-sequencing analysis revealed that *Oct-TyrR* transcript levels were elevated not only in the pupal CNS and eye but also in the salivary glands (Figure 8). Although other OARs exhibited similar expression profiles, it remains to be elucidated why certain OARs impact pupal survival while others do not. Specifically, the high abundance of *Oct-TyrR* transcripts in prepupal salivary glands, which is a major secretory organ that undergoes autophagy during the early phases of metamorphosis, suggests a critical developmental function for OA signaling.

A role for Oct-TyrR in locomotion was first described in *honoka* mutants, which exhibited reduced excitatory junctional potential (EJP) amplitudes in body-wall muscles (Kutsukake et al., 2000; Nagaya et al., 2002). Later, expression was detected in the CNS, MNs, and at the neuromuscular junction (NMJ; Stowers, 2011; Figure 4). Our analysis of locomotion behavior in Trojan *Oct-TyrR* revealed no impairment compared to the control (Figure 3C), consistent with previous data from *honoka* larvae (Schützler et al., 2019). Although we reported a hyperactivity phenotype in *honoka* (Selcho et al., 2012), a direct comparison between these two studies is difficult; this discrepancy arises not only from the use of different control lines but also from distinct tracking methodologies, specifically automated (Schützler et al., 2019) versus manual tracking (Selcho et al., 2012). Consequently, further research is needed to clarify the role of Oct-TyrR in larval locomotion.

### The OAR system

OA plays a role in various mechanisms spanning from embryogenesis through larval development to adulthood. A substantial body of literature has already identified multiple OA receptors involved in the same biological processes (Figure 1). Here, we observed a phenotypic overlap among three OARs (*Octβ1R*, *Octβ3R*, and *Oct-TyrR*) involved in embryonic development and/or larval hatching. Embryogenesis comprises 17 stages, with organ differentiation beginning at stage 13 and continuing through stage 16. During this phase, elevated *Octβ1R* transcript levels have been reported in the CNS and PNS (Berkeley Drosophila Genome Project; Tomancak et al., 2002; Tomancak et al., 2007). Similarly, during stages 15–17, Ohhara et al. (2012) observed increased mRNA levels of both *Octβ1R* and *Octβ3R* in the CNS as well as the whole embryo. Unfortunately, embryonic transcript data for *Oct-TyrR* are currently unavailable. Although the specific roles of Octβ1R, Octβ3R, and Oct-TyrR during embryogenesis remain to be fully investigated, their elevated embryonic expression levels coupled with the reduced hatching rates observed here warrant further functional studies. Alternatively, maternal effects cannot be ruled out. Embryos developing from eggs produced by a mutant mother, which lack maternal contribution, could account for the observed offspring lethality. Future experiments utilizing heterozygous mothers will be necessary to distinguish between zygotic and maternal effects, or a combination of both, on embryonic development. In conclusion, our data demonstrate a novel role for Octβ1R, Octβ3R, and Oct-TyrR in ensuring overall developmental viability.

Furthermore, consistent with previous data, anatomical mapping revealed an overlap of OARs across the third-instar larva, pupa, and adult stages (Figures 4–6, 8; Supplementary Table 11). However, whether distinct OARs are co-expressed within identical cells remains to be confirmed. Implementing a combination of different protein tags, such as Venus (Kondo et al., 2020), could provide deeper insight into co-expression and subcellular receptor localization within individual cells.

One experimental approach to understanding the functional implications of overlapping OAR labeling was to utilize chemosensory assays. The sensory system in the larval PCE exhibited distinct labeling for OARs in the DO, TO, and VO; however, behavioral tests did not suggest a direct involvement of OA. The similarities in OAR labeling patterns within the same functional unit for olfactory or gustatory perception may indicate that other OARs compensate for the absence of receptors in single OAR lines. This redundancy is particularly plausible given that downstream signaling is conserved among GPCRs, and certain OARs exhibit similar G-protein coupling (Table 1). Since OA is involved in various mechanisms crucial for survival, a redundant system wherein one receptor can compensate for the loss of another would be less vulnerable to interference. For instance, ectopic expression *of Octβ1R* in the oviduct epithelium has been shown to substitute for *Octβ2R* and restore ovulation in *Octβ2R^f05679^* females (Lim et al., 2014). However, whether such a compensatory effect occurs in the chemosensory system remains to be clarified, as does whether it operates at the level of the functional unit or within individual cells. Alternatively, the diverse downstream pathways of OARs suggest a potential modulatory mechanism. For instance, OA signaling via Oamb, Octβ1R, and Octβ2R contributes to synaptic plasticity at the NMJ in a modulatory manner (Koon et al., 2011; Koon and Budnik, 2012; Bakshinska et al., 2025; Blaum et al., 2025). Ultimately, it remains to be elucidated whether the OA system functions in a redundant or modulatory mode, or perhaps both, depending on the cell type. Future investigations targeting these roles could utilize double and triple mutants in developmental, behavioral, and physiological assays.

## Data Availability

The datasets presented in this study can be found in online repositories. The names of the repository/repositories and accession number(s) can be found in the article/Supplementary material.

## Footnotes

1
https://imagej.net/ij

2
https://www.ebi.ac.uk

## References

[B1] Andrews J. C. Fernandez M. P. Yu Q. Leary G. P. Leung A. K. Kavanaugh M. P. et al. (2014). Octopamine neuromodulation regulates Gr32a-linked aggression and courtship pathways in drosophila males. *PLoS Genet.* 10:e1004356. 10.1371/journal.pgen.1004356 24852170 PMC4031044

[B2] Apostolopoulou A. A. Widmann A. Rohwedder A. Pfitzenmaier J. E. Thum A. S. (2013). Appetitive associative olfactory learning in drosophila larvae. *J. Vis. Exp.* 18:4334. 10.3791/4334-v PMC3601210 23438816

[B3] Avila F. W. Bloch Qazi M. C. Rubinstein C. D. Wolfner M. F. (2012). A requirement for the neuromodulators octopamine and tyramine in *Drosophila melanogaster* female sperm storage. *Proc. Natl. Acad. Sci. U S A.* 109 4562–4567. 10.1073/pnas.1117689109 22393023 PMC3311333

[B4] Babski H. Codianni M. Bhandawat V. (2024). Octopaminergic descending neurons in Drosophila: Connectivity, tonic activity and relation to locomotion. *Heliyon* 10:e29952. 10.1016/j.heliyon.2024.e29952 38698992 PMC11064449

[B5] Baier A. Wittek B. Brembs B. (2002). *Drosophila* as a new model organism for the neurobiology of aggression? *J. Exp. Biol.* 205 1233–1240. 10.1242/jeb.205.9.1233 11948200

[B6] Bakker K. (1959). Feeding period, growth, and pupation in larvae of *Drosophila melanogaster*. *Entomol. Exp. Appl.* 2 171–186. 10.1111/j.1570-7458.1959.tb00432.x

[B7] Bakker K. (1961). An analysis of factors which determine success in competition for food among larvae of *Drosophila Melanogaster*. *Arch. Néerlandaises Zool.* 14 200–281. 10.1163/036551661X00061

[B8] Bakshinska D. Liu W. Y. Schultz R. Stowers R. S. Hoagland A. Cypranowska C. et al. (2025). Synapse-specific catecholaminergic modulation of neuronal glutamate release. *Proc. Natl. Acad. Sci. U S A.* 122:e2420496121. 10.1073/pnas.2420496121 39793084 PMC11725921

[B9] Balfanz S. Strunker T. Frings S. Baumann A. (2005). A family of octapamine receptors that specifically induce cyclic AMP production or Ca2+ release in *Drosophila melanogaster*. *J. Neurochem.* 93 440–451. 10.1111/j.1471-4159.2005.03034.x 15816867

[B10] Berger M. Fraatz M. Auweiler K. Dorn K. El Khadrawe T. Scholz H. (2024). Octopamine integrates the status of internal energy supply into the formation of food-related memories. *Elife* 12:R88247. 10.7554/eLife.88247 38655926 PMC11042807

[B11] Bhatt P. K. Neckameyer W. S. (2013). Functional analysis of the larval feeding circuit in *Drosophila*. *J. Vis. Exp.* 81:e51062. 10.3791/51062 24300174 PMC3991465

[B12] Blaum N. Ghelani T. Gotz T. W. B. Chronister K. S. Bengochea M. Ceresnova L. et al. (2025). Monoamine-induced diacylglycerol signaling rapidly accumulates Unc13 in nanoclusters for fast presynaptic potentiation. *Proc. Natl. Acad. Sci. U S A.* 122:e2514151122. 10.1073/pnas.2514151122 40833403 PMC12403152

[B13] Bodmer R. Jan Y. N. (1987). Morphological characterization of the embryonic peripheral neurons in *Drosophila*. *Roux’s Arch. Dev. Biol.* 196 69–77. 10.1007/BF00402027 28305460

[B14] Boivin J. C. Zhao Y. Q. Zhu J. Dakin J. T. Ning J. Ohyama T. (2026). A positive feedback loop between sensory and octopaminergic neurons underlies nociceptive plasticity in Drosophila larvae. *PLoS Genet.* 22:e1012122. 10.1371/journal.pgen.1012122 42048383 PMC13152156

[B15] Bragina J. V. Goncharova A. A. Besedina N. G. Danilenkova L. V. Kamysheva E. A. Kamyshev N. G. (2025). Genetic control of social experience-dependent changes in locomotor activity in drosophila melanogaster males. *Arch. Insect. Biochem. Physiol.* 118:e70022. 10.1002/arch.70022 39966324

[B16] Branch A. Zhang Y. Shen P. (2017). Genetic and neurobiological analyses of the noradrenergic-like system in vulnerability to sugar overconsumption using a drosophila model. *Sci. Rep.* 7:17642. 10.1038/s41598-017-17760-w 29247240 PMC5732301

[B17] Brembs B. Christiansen F. Pfluger H. J. Duch C. (2007). Flight initiation and maintenance deficits in flies with genetically altered biogenic amine levels. *J. Neurosci.* 27 11122–11131. 10.1523/JNEUROSCI.2704-07.2007 17928454 PMC6672854

[B18] Burke C. J. Huetteroth W. Owald D. Perisse E. Krashes M. J. Das G. et al. (2012). Layered reward signalling through octopamine and dopamine in *Drosophila*. *Nature* 492 433–437. 10.1038/nature11614 23103875 PMC3528794

[B19] Busch S. Tanimoto H. (2010). Cellular configuration of single octopamine neurons in Drosophila. *J. Comp. Neurol.* 518 2355–2364. 10.1002/cne.22337 20437532

[B20] Busch S. Selcho M. Ito K. Tanimoto H. (2009). A map of octopaminergic neurons in the Drosophila brain. *J. Comp. Neurol.* 513 643–667. 10.1002/cne.21966 19235225

[B21] Caldwell J. C. Miller M. M. Wing S. Soll D. R. Eberl D. F. (2003). Dynamic analysis of larval locomotion in *Drosophila* chordotonal organ mutants. *Proc. Natl. Acad. Sci. U S A.* 100 16053–16058. 10.1073/pnas.2535546100 14673076 PMC307691

[B22] Certel S. J. Leung A. Lin C. Y. Perez P. Chiang A. S. Kravitz E. A. (2010). Octopamine neuromodulatory effects on a social behavior decision-making network in *Drosophila* males. *PLoS One* 5:e13248. 10.1371/journal.pone.0013248 20967276 PMC2953509

[B23] Certel S. Savella M. G. Schlegel D. C. F. Kravitz E. A. (2007). Modulation of *Drosophila* male behavioral choice. *Proc. Natl. Acad. Sci. U S A.* 104 4706–4711. 10.1073/pnas.0700328104 17360588 PMC1810337

[B24] Chatwin H. M. Rudling J. E. Patel D. Reale V. Evans P. D. (2003). Site-directed mutagenesis studies on the *Drosophila* octopamine/tyramine receptor. *Insect. Biochem. Mol. Biol.* 33 173–184. 10.1016/s0965-1748(02)00188-1 12535676

[B25] Classen G. Scholz H. (2018). Octopamine shifts the behavioral response from indecision to approach or aversion in drosophila melanogaster. *Front. Behav. Neurosci.* 12:131. 10.3389/fnbeh.2018.00131 30018540 PMC6037846

[B26] Cobb M. (1999). What and how do maggots smell? *Biol. Rev.* 74 425–459. 10.1017/S0006323199005393

[B27] Cole S. H. Carney G. E. McClung C. A. Willard S. S. Taylor B. J. Hirsh J. (2005). Two functional but noncomplementing Drosophila tyrosine decarboxylase genes: Distinct roles for neural tyramine and octopamine in female fertility. *J. Biol. Chem.* 280 14948–14955. 10.1074/jbc.M414197200 15691831

[B28] Crocker A. Sehgal A. (2008). Octopamine regulates sleep in *Drosophila* through protein kinase A-dependent mechanisms. *J. Neurosci.* 28 9377–9385. 10.1523/JNEUROSCI.3072-08a.2008 18799671 PMC2742176

[B29] Crocker A. Shahidullah M. Levitan I. B. Sehgal A. (2010). Identification of a neural circuit that underlies the effects of octopamine on sleep:wake behavior. *Neuron* 65 670–681. 10.1016/j.neuron.2010.01.032 20223202 PMC2862355

[B30] Damrau C. Toshima N. Tanimura T. Brembs B. Colomb J. (2018). Octopamine and tyramine contribute separately to the counter-regulatory response to sugar deficit in drosophila. *Front. Syst. Neurosci.* 11:100. 10.3389/fnsys.2017.00100 29379421 PMC5775261

[B31] David J. C. Coulon J.-F. (1985). Octopamine in invertebrates and vertebrates. A review. *Prog. Neurobiol.* 24 141–185. 10.1016/0301-0082(85)90009-7 2863854

[B32] Deady L. D. Sun J. (2015). A follicle rupture assay reveals an essential role for follicular adrenergic signaling in *Drosophila* ovulation. *PLoS Genet.* 11:e1005604. 10.1371/journal.pgen.1005604 26473732 PMC4608792

[B33] Deng B. Li Q. Liu X. Cao Y. Li B. Qian Y. et al. (2019). Chemoconnectomics: Mapping chemical transmission in *Drosophila*. *Neuron* 101 876–893.e874. 10.1016/j.neuron.2019.01.045 30799021

[B34] Diao F. White B. H. (2012). A novel approach for directing transgene expression in *Drosophila*: T2A-Gal4 in-frame fusion. *Genetics* 190 1139–1144. 10.1534/genetics.111.136291 22209908 PMC3296248

[B35] Diao F. Ironfield H. Luan H. Diao F. Shropshire W. C. Ewer J. et al. (2015). Plug-and-play genetic access to *Drosophila* cell types using exchangeable exon cassettes. *Cell. Rep.* 10 1410–1421. 10.1016/j.celrep.2015.01.059 25732830 PMC4373654

[B36] El-Kholy S. E. Afifi B. El-Husseiny I. Seif A. (2022). Octopamine signaling via OctalphaR is essential for a well-orchestrated climbing performance of adult Drosophila melanogaster. *Sci. Rep.* 12:14024. 10.1038/s41598-022-18203-x 35982189 PMC9388497

[B37] El-Kholy S. Stephano F. Li Y. Bhandari A. Fink C. Roeder T. (2015). Expression analysis of octopamine and tyramine receptors in *Drosophila*. *Cell Tissue Res.* 361 669–684. 10.1007/s00441-015-2137-4 25743690

[B38] Erion R. DiAngelo J. R. Crocker A. Sehgal A. (2012). Interaction between sleep and metabolism in Drosophila with altered octopamine signaling. *J. Biol. Chem.* 287 32406–32414. 10.1074/jbc.M112.360875 22829591 PMC3463357

[B39] Farooqui T. (2012). Review of octopamine in insect nervous systems. *Open Access. Insect. Physiol* 4, 1–17. 10.2147/oaip.S20911

[B40] Ferreira Castro A. Baltruschat L. Sturner T. Bahrami A. Jedlicka P. Tavosanis G. et al. (2020). Achieving functional neuronal dendrite structure through sequential stochastic growth and retraction. *eLife* 9:e60920. 10.7554/eLife.60920 33241995 PMC7837678

[B41] Fishilevich E. Domingos A. I. Asahina K. Naef F. Vosshall L. B. Louis M. (2005). Chemotaxis behavior mediated by single larval olfactory neurons in Drosophila. *Curr. Biol.* 15 2086–2096. 10.1016/j.cub.2005.11.016 16332533

[B42] Fox L. E. Soll D. R. Wu C. F. (2006). Coordination and modulation of locomotion pattern generators in *Drosophila* larvae: Effects of altered biogenic amine levels by the tyramine beta hydroxlyase mutation. *J. Neurosci.* 26 1486–1498. 10.1523/JNEUROSCI.4749-05.2006 16452672 PMC2673197

[B43] Franke U. S. Großjohann A. Aurich S. Kohler I. Lamberty M. Granato S. et al. (2026). Selective octopaminergic tuning of mushroom body circuits during memory formation. *Proc. Natl. Acad. Sci. U S A.* 123:e2517403123. 10.1073/pnas.2517403123 41980099 PMC13099586

[B44] Gerber B. Stocker R. F. (2007). The *Drosophila* larva as a model for studying chemosensation and chemosensory learning: A review. *Chem. Senses* 32 65–89. 10.1093/chemse/bjl030 17071942

[B45] Gershow M. Berck M. Mathew D. Luo L. Kane E. A. Carlson J. R. et al. (2012). Controlling airborne cues to study small animal navigation. *Nat. Methods* 9 290–296. 10.1038/nmeth.1853 22245808 PMC3513333

[B46] Gjorgjieva J. Berni J. Evers J. F. Eglen S. J. (2013). Neural circuits for peristaltic wave propagation in crawling Drosophila larvae: Analysis and modeling. *Front. Comput. Neurosci.* 7:24. 10.3389/fncom.2013.00024 23576980 PMC3616270

[B47] Gomez-Marin A. Stephens G. J. Louis M. (2011). Active sampling and decision making in *Drosophila* chemotaxis. *Nat. Commun.* 2:441. 10.1038/ncomms1455 21863008 PMC3265367

[B48] Gonzalez F. Witzgall P. Walker W. B. (2016). Protocol for heterologous expression of insect odourant receptors in *Drosophila*. *Front. Ecol. Evol.* 4:24. 10.3389/fevo.2016.00024

[B49] Großjohann A. Richter V. Reinhardt F. Hahmann M. Badelt R. Kinnigkeit J. et al. (2026). Octopamine receptors at a glance: From expression and anatomical maps to their role in development and behavior in the *Drosophila melanogaster* larva. *bioRxiv [Preprint]* 10.64898/2026.05.05.722892

[B50] Han K.-A. Millar N. S. Davis R. L. (1998). A novel octopamine receptor with preferential expression in *Drosophila* mushroom bodies. *J. Neurosci.* 18 3650–3658. 10.1523/JNEUROSCI.18-10-03650.1998 9570796 PMC6793132

[B51] He L. Gulyanon S. Mihovilovic Skanata M. Karagyozov D. Heckscher E. S. Krieg M. et al. (2019). Direction selectivity in *Drosophila* proprioceptors requires the mechanosensory channel Tmc. *Curr. Biol.* 29 945–956 e943. 10.1016/j.cub.2019.02.025 30853433 PMC6884367

[B52] Heckscher E. S. Zarin A. A. Faumont S. Clark M. Q. Manning L. Fushiki A. et al. (2015). Even-Skipped^+^ interneurons are core components of a sensorimotor circuit that maintains left-right symmetric muscle contraction amplitude. *Neuron* 88 314–329. 10.1016/j.neuron.2015.09.009 26439528 PMC4619170

[B53] Hoff M. Balfanz S. Ehling P. Gensch T. Baumann A. (2011). A single amino acid residue controls Ca^2+^ signaling by an octopamine receptor from *Drosophila melanogaster*. *FASEB J* 25 2484–2491. 10.1096/fj.11-180703 21478261 PMC3114530

[B54] Hooper J. E. (1986). Homeotic gene function in the muscles of *Drosophila* larvae. *EMBO J.* 5 2321–3229. 10.1002/j.1460-2075.1986.tb04500.x 16453704 PMC1167116

[B55] Hoyer S. C. Eckart A. Herrel A. Zars T. Fischer S. A. Hardie S. L. et al. (2008). Octopamine in male aggression of *Drosophila*. *Curr. Biol.* 18 159–167. 10.1016/j.cub.2007.12.052 18249112

[B56] Hu C. Petersen M. Hoyer N. Spitzweck B. Tenedini F. Wang D. et al. (2017). Sensory integration and neuromodulatory feedback facilitate *Drosophila* mechanonociceptive behavior. *Nat. Neurosci.* 20 1085–1095. 10.1038/nn.4580 28604684 PMC5931224

[B57] Huang Y. Ainsley J. A. Reijmers L. G. Jackson F. R. (2013). Translational profiling of clock cells reveals circadianly synchronized protein synthesis. *PLoS Biol.* 11:e1001703. 10.1371/journal.pbio.1001703 24348200 PMC3864454

[B58] Hückesfeld S. Schlegel P. Miroschnikow A. Schoofs A. Zinke I. Haubrich A. N. et al. (2021). Unveiling the sensory and interneuronal pathways of the neuroendocrine connectome in *Drosophila*. *eLife* 10:e65745. 10.7554/eLife.65745 34085637 PMC8177888

[B59] Huetteroth W. Perisse E. Lin S. Klappenbach M. Burke C. Waddell S. (2015). Sweet taste and nutrient value subdivide rewarding dopaminergic neurons in *Drosophila*. *Curr. Biol.* 25 751–758. 10.1016/j.cub.2015.01.036 25728694 PMC4372253

[B60] Imura E. Shimada-Niwa Y. Nishimura T. Huckesfeld S. Schlegel P. Ohhara Y. et al. (2020). The corazonin-PTTH neuronal axis controls systemic body growth by regulating basal ecdysteroid biosynthesis in *Drosophila melanogaster*. *Curr. Biol.* 30 2156–2165.e2155. 10.1016/j.cub.2020.03.050 32386525

[B61] Ito K. Hotta Y. (1992). Proliferation pattern of postembryonic neuroblasts in the brain of Drosophila melanogaster. *Dev. Biol.* 149 134–148. 10.1016/0012-1606(92)90270-Q 1728583

[B62] Jan Y. N. Jan L. Y. (1993). “The peripheral nervous system,” in *The Development of Drosophila Melanogaster*, ed. Bate M. (Cold Spring Harbor, NY: Cold Spring Harbor Laboratory Press), 1207–1244.

[B63] Jetti S. K. Crane A. B. Akbergenova Y. Aponte-Santiago N. A. Cunningham K. L. Whittaker C. A. et al. (2023). Molecular logic of synaptic diversity between *Drosophila* tonic and phasic motoneurons. *Neuron* 111 3554–3569.e3557. 10.1016/j.neuron.2023.07.019 37611584

[B64] Kim D. Alvarez M. Lechuga L. M. Louis M. (2017). Species-specific modulation of food-search behavior by respiration and chemosensation in Drosophila larvae. *Elife* 6:e27057. 10.7554/eLife.27057 28871963 PMC5584988

[B65] Kim Y. C. Lee H. G. Lim J. Han K. A. (2013). Appetitive learning requires the alpha1-like octopamine receptor OAMB in the *Drosophila* mushroom body neurons. *J. Neurosci.* 33 1672–1677. 10.1523/JNEUROSCI.3042-12.2013 23345239 PMC5634613

[B66] Kondo S. Takahashi T. Yamagata N. Imanishi Y. Katow H. Hiramatsu S. et al. (2020). Neurochemical organization of the *Drosophila* brain visualized by endogenously tagged neurotransmitter receptors. *Cell. Rep.* 30 284–297.e285. 10.1016/j.celrep.2019.12.018 31914394

[B67] Koon A. C. Budnik V. (2012). Inhibitory control of synaptic and behavioral plasticity by octopaminergic signaling. *J. Neurosci.* 32 6312–6322. 10.1523/JNEUROSCI.6517-11.2012 22553037 PMC3371232

[B68] Koon A. C. Ashley J. Barria R. DasGupta S. Brain R. Waddell S. et al. (2011). Autoregulatory and paracrine control of synaptic and behavioral plasticity by octopaminergic signaling. *Nat. Neurosci.* 14 190–199. 10.1038/nn.2716 21186359 PMC3391700

[B69] Kula-Eversole E. Nagoshi E. Shang Y. Rodriguez J. Allada R. Rosbash M. (2010). Surprising gene expression patterns within and between PDF-containing circadian neurons in Drosophila. *Proc. Natl. Acad. Sci. U S A.* 107 13497–13502. 10.1073/pnas.1002081107 20624977 PMC2922133

[B70] Kutsukake M. Komatsu A. Yamamoto D. Ishiwa-Chigusa S. (2000). A tyramine receptor gene mutation causes a defective olfactory behavior in *Drosophila melanogaster*. *Gene* 245 31–42. 10.1016/S0378-1119(99)00569-7 10713442

[B71] Lee H. G. Rohila S. Han K. A. (2009). The octopamine receptor OAMB mediates ovulation via Ca2+/calmodulin-dependent protein kinase II in the *Drosophila* oviduct epithelium. *PLoS One* 4:e0004716. 10.1371/journal.pone.0004716 19262750 PMC2650798

[B72] Lee H.-G. Seong C.-S. Kim Y.-C. Davis R. L. Han K.-A. (2003). Octopamine receptor OAMB is required for ovulation in *Drosophila melanogaster*. *Dev. Biol.* 264 179–190. 10.1016/j.ydbio.2003.07.018 14623240

[B73] Lee S. H. Lee H. Y. Lee E. J. Khang D. Min K. J. (2015). Effects of carbon nanofiber on physiology of Drosophila. *Int. J. Nanomed.* 10 3687–3697. 10.2147/IJN.S82637 26056448 PMC4445953

[B74] Li K. Tsukasa Y. Kurio M. Maeta K. Tsumadori A. Baba S. et al. (2023). Belly roll, a GPI-anchored Ly6 protein, regulates *Drosophila melanogaster* escape behaviors by modulating the excitability of nociceptive peptidergic interneurons. *eLife* 12:e83856. 10.7554/eLife.83856 37309249 PMC10264074

[B75] Li Y. Fink C. El-Kholy S. Roeder T. (2015). The octopamine receptor octß2R is essential for ovulation and fertilization in the fruit fly *Drosophila melanogaster*. *Arch. Insect. Biochem. Physiol.* 88 168–178. 10.1002/arch.21211 25353988

[B76] Li Y. Hoffmann J. Li Y. Stephano F. Bruchhaus I. Fink C. et al. (2016). Octopamine controls starvation resistance, life span and metabolic traits in Drosophila. *Sci. Rep.* 6:35359. 10.1038/srep35359 27759117 PMC5069482

[B77] Lim J. Sabandal P. R. Fernandez A. Sabandal J. M. Lee H. G. Evans P. et al. (2014). The octopamine receptor Octbeta2R regulates ovulation in *Drosophila melanogaster*. *PLoS One* 9:e104441. 10.1371/journal.pone.0104441 25099506 PMC4123956

[B78] Liu Y. Hasegawa E. Nose A. Zwart M. F. Kohsaka H. (2023). Synchronous multi-segmental activity between metachronal waves controls locomotion speed in Drosophila larvae. *eLife* 12:e83328. 10.7554/eLife.83328 37551094 PMC10409504

[B79] Louis M. Huber T. Benton R. Sakmar T. P. Vosshall L. B. (2008). Bilateral olfactory sensory input enhances chemotaxis behavior. *Nat. Neurosci.* 11 187–199. 10.1038/nn2031 18157126

[B80] Lu S. Qian C. S. Grueber W. B. (2024). Mechanisms of gas sensing by internal sensory neurons in *Drosophila* larvae. *bioRxiv [Preprint]* 10.1101/2024.01.20.576342

[B81] Luo J. Lushchak O. V. Goergen P. Williams M. J. Nassel D. R. (2014). *Drosophila* insulin-producing cells are differentially modulated by serotonin and octopamine receptors and affect social behavior. *PLoS One* 9:e99732. 10.1371/journal.pone.0099732 24923784 PMC4055686

[B82] Ma Z. Stork T. Bergles D. E. Freeman M. R. (2016). Neuromodulators signal through astrocytes to alter neural circuit activity and behaviour. *Nature* 539 428–432. 10.1038/nature20145 27828941 PMC5161596

[B83] Manjila S. B. Kuruvilla M. Ferveur J. F. Sane S. P. Hasan G. (2019). Extended flight bouts require disinhibition from GABAergic mushroom body neurons. *Curr. Biol.* 29 283–293.e285. 10.1016/j.cub.2018.11.070 30612904

[B84] Maqueira B. Chatwin H. Evans P. D. (2005). Identification and characterization of a novel family of *Drosophila* beta-adrenergic-like octopamine G-protein coupled receptors. *J. Neurochem.* 94 547–560. 10.1111/j.1471-4159.2005.03251.x 15998303

[B85] Mezheritskiy M. I. Vorontsov D. D. Dyakonova V. E. Zakharov I. S. (2024). Behavioral functions of octopamine in adult insects under stressful conditions. *Biol. Bull. Rev.* 14 535–547. 10.1134/s2079086424700014

[B86] Monastirioti M. (2003). Distinct octopamine cell population residing in the CNS abdominal ganglion controls ovulation in *Drosophila melanogaster*. *Dev. Biol.* 264 38–49. 10.1016/j.ydbio.2003.07.019 14623230

[B87] Monastirioti M. Gorczyca M. Rapus J. Eckert M. White K. Budnik V. (1995). Octopamine immunoreactivity in the fruit fly *Drosophila melanogaster*. *J. Comp. Neurol.* 356 275–287. 10.1002/cne.903560210 7629319 PMC4664080

[B88] Monastirioti M. Linn C. E. White K. (1996). Characterization of *Drosophila* tyramine B-hydroxylase gene and isolation of mutant flies lacking octopamine. *J. Neurosci.* 16 3900–3911. 10.1523/JNEUROSCI.16-12-03900.1996 8656284 PMC6578608

[B89] Nagaya Y. Kutsukake M. Chigusa S. I. Komatsu A. (2002). A trace amine, tyramine, functions as a neuromodulator in *Drosophila melanogaster*. *Neurosci. Lett.* 329 324–328. 10.1016/S0304-3940(02)00596-7 12183041

[B90] Nakagawa H. Maehara S. Kume K. Ohta H. Tomita J. (2022). Biological functions of alpha2-adrenergic-like octopamine receptor in *Drosophila melanogaster*. *Genes Brain Behav.* 21:e12807. 10.1111/gbb.12807 35411674 PMC9744561

[B91] Ohhara Y. Kayashima Y. Hayashi Y. Kobayashi S. Yamakawa-Kobayashi K. (2012). Expression of β-adrenergic-like octopamine receptors during *Drosophila* development. *Zool. Sci.* 29 83–89. 10.2108/zsj.29.83 22303848

[B92] Ohhara Y. Shimada-Niwa Y. Niwa R. Kayashima Y. Hayashi Y. Akagi K. et al. (2015). Autocrine regulation of ecdysone synthesis by beta3-octopamine receptor in the prothoracic gland is essential for *Drosophila* metamorphosis. *Proc. Natl. Acad. Sci. U S A.* 112 1452–1457. 10.1073/pnas.1414966112 25605909 PMC4321272

[B93] Ohnishi S. (1979). Relationship between larval feeding behavior and viability in *Drosophila melanogaster* and *D. simulans*. *Behav. Genet.* 9 129–134. 10.1007/BF01074332 120743

[B94] Orsted M. Hoffmann A. A. Sverrisdottir E. Nielsen K. L. Kristensen T. N. (2019). Genomic variation predicts adaptive evolutionary responses better than population bottleneck history. *PLoS Genet.* 15:e1008205. 10.1371/journal.pgen.1008205 31188830 PMC6590832

[B95] Öztürk-Çolak A. Marygold S. J. Antonazzo G. Attrill H. Goutte-Gattat D. Jenkins V. K. et al. (2024). FlyBase: Updates to the *Drosophila* genes and genomes database. *Genetics* 227:iyad211. 10.1093/genetics/iyad211 38301657 PMC11075543

[B96] Pauls D. Blechschmidt C. Frantzmann F. El Jundi B. Selcho M. (2018). A comprehensive anatomical map of the peripheral octopaminergic/tyraminergic system of *Drosophila melanogaster*. *Sci. Rep.* 8:15314. 10.1038/s41598-018-33686-3 30333565 PMC6192984

[B97] Pauls D. Selcho M. Gendre N. Stocker R. F. Thum A. S. (2010). *Drosophila* larvae establish appetitive olfactory memories via mushroom body neurons of embryonic origin. *J. Neurosci.* 30 10655–10666. 10.1523/JNEUROSCI.1281-10.2010 20702697 PMC6634688

[B98] Pauls D. Selcho M. Raderscheidt J. Amatobi K. M. Fekete A. Krischke M. et al. (2021). Endocrine signals fine-tune daily activity patterns in Drosophila. *Curr. Biol.* 31 4076–4087.e4075. 10.1016/j.cub.2021.07.002 34329588

[B99] Pauls D. von Essen A. Lyutova R. van Giesen L. Rosner R. Wegener C. et al. (2015). Potency of transgenic effectors for neurogenetic manipulation in Drosophila larvae. *Genetics* 199 25–37. 10.1534/genetics.114.172023 25359929 PMC4286689

[B100] Qi Y. X. Xu G. Gu G. X. Mao F. Ye G. Y. Liu W. et al. (2017). A new *Drosophila* octopamine receptor responds to serotonin. *Insect. Biochem. Mol. Biol.* 90 61–70. 10.1016/j.ibmb.2017.09.010 28942992

[B101] Ramaekers A. Magnenat E. Marin E. C. Gendre N. Jefferis G. S. Luo L. et al. (2005). Glomerular maps without cellular redundancy at successive levels of the Drosophila larval olfactory circuit. *Curr. Biol.* 15 982–992. 10.1016/j.cub.2005.04.032 15936268

[B102] Reyes M. Lee Y. S. Ali M. M. Sundaramurthi P. Dhungana N. Nguyen A. et al. (2026). Octopamine regulates neural circuits in the mushroom body and central complex, influencing sleep and arousal. *iScience* 29:115564. 10.1016/j.isci.2026.115564 42058909 PMC13122709

[B103] Rezaval C. Nojima T. Neville M. C. Lin A. C. Goodwin S. F. (2014). Sexually dimorphic octopaminergic neurons modulate female postmating behaviors in *Drosophila*. *Curr. Biol.* 24 725–730. 10.1016/j.cub.2013.12.051 24631243 PMC7613681

[B104] Richter V. Rist A. Kislinger G. Laumann M. Schoofs A. Miroschnikow A. et al. (2023). Morphology and ultrastructure of external sense organs of *Drosophila* larvae. *eLife* 12:R91155. 10.7554/eLife.91155.1 PMC12169854 40522083

[B105] Rist A. Thum A. S. (2017). A map of sensilla and neurons in the taste system of *Drosophila* larvae. *J. Comp. Neurol.* 525 3865–3889. 10.1002/cne.24308 28842919

[B106] Robb S. Cheek T. R. Hannan F. L. Hall L. M. Midgley J. M. Evans P. D. (1994). Agonist-specific coupling of a cloned *Drosophila* octopamine/tyramine receptor to multiple second messenger systems. *EMBO J.* 13 1325–1330. 10.1002/j.1460-2075.1994.tb06385.x 8137817 PMC394948

[B107] Roeder T. (2005). Tyramine and octopamine: Ruling behavior and metabolism. *Annu. Rev. Entomol.* 50 447–477. 10.1146/annurev.ento.50.071803.130404 15355245

[B108] Roeder T. (2020). The control of metabolic traits by octopamine and tyramine in invertebrates. *J. Exp. Biol.* 223:jeb194282. 10.1242/jeb.194282 32238440

[B109] Rohrbach E. W. Asuncion J. D. Meera P. Kralovec M. Deshpande S. A. Schweizer F. E. et al. (2024). Heterogeneity in the projections and excitability of tyraminergic/octopaminergic neurons that innervate the *Drosophila* reproductive tract. *Front. Mol. Neurosci.* 17:1374896. 10.3389/fnmol.2024.1374896 39156129 PMC11327148

[B110] Rohwedder A. Pfitzenmaier J. E. Ramsperger N. Apostolopoulou A. A. Widmann A. Thum A. S. (2012). Nutritional value-dependent and nutritional value-independent effects on *Drosophila melanogaster* larval behavior. *Chem. Senses* 37 711–721. 10.1093/chemse/bjs055 22695795

[B111] Sabandal J. M. Sabandal P. R. Kim Y. C. Han K. A. (2020). Concerted actions of octopamine and dopamine receptors drive olfactory learning. *J. Neurosci.* 40 4240–4250. 10.1523/JNEUROSCI.1756-19.2020 32277043 PMC7244198

[B112] Saraswati S. Fox L. E. Soll D. R. Wu C. F. (2004). Tyramine and octopamine have opposite effects on the locomotion of *Drosophila* larvae. *J. Neurobiol.* 58 425–441. 10.1002/neu.10298 14978721

[B113] Saudou F. Amlaiky N. Plassat J.-L. Borrelli E. Hen R. (1990). Cloning and characterization of a *Drosophila* tyramine receptor. *EMBO J.* 9 3611–3617. 10.1002/j.1460-2075.1990.tb07572.x 2170118 PMC1326448

[B114] Schleyer M. Saumweber T. Nahrendorf W. Fischer B. von Alpen D. Pauls D. et al. (2011). A behavior-based circuit model of how outcome expectations organize learned behavior in larval *Drosophila*. *Learn. Mem.* 18 639–653. 10.1101/lm.2163411 21946956

[B115] Schneider A. Ruppert M. Hendrich O. Giang T. Ogueta M. Hampel S. et al. (2012). Neuronal basis of innate olfactory attraction to ethanol in *Drosophila*. *PLoS One* 7:e52007. 10.1371/journal.pone.0052007 23284851 PMC3527413

[B116] Scholz H. Ramond J. Singh C. M. Heberlein U. (2000). Functional ethanol tolerance in *Drosophila*. *Neuron* 28 261–271. 10.1016/S0896-6273(00)00101-X 11086999

[B117] Schoofs A. Niederegger S. van Ooyen A. Heinzel H. G. Spiess R. (2010). The brain can eat: Establishing the existence of a central pattern generator for feeding in third instar larvae of *Drosophila virilis* and *Drosophila melanogaster*. *J. Insect. Physiol.* 56 695–705. 10.1016/j.jinsphys.2009.12.008 20074578

[B118] Schou M. F. (2013). Fast egg collection method greatly improves randomness of egg sampling in Drosophila melanogaster. *Fly* 7 44–46. 10.4161/fly.22758 23247611 PMC3660287

[B119] Schretter C. E. Vielmetter J. Bartos I. Marka Z. Marka S. Argade S. et al. (2018). A gut microbial factor modulates locomotor behaviour in Drosophila. *Nature* 563 402–406. 10.1038/s41586-018-0634-9 30356215 PMC6237646

[B120] Schützler N. Girwert C. Hugli I. Mohana G. Roignant J. Y. Ryglewski S. et al. (2019). Tyramine action on motoneuron excitability and adaptable tyramine/octopamine ratios adjust Drosophila locomotion to nutritional state. *Proc. Natl. Acad. Sci. U S A.* 116 3805–3810. 10.1073/pnas.1813554116 30808766 PMC6397572

[B121] Schwaerzel M. Monastirioti M. Scholz H. Friggi-Grelin F. Birman S. Heisenberg M. (2003). Dopamine and octopamine differentiate between aversive and appetitive olfactory memories in *Drosophila*. *J. Neurosci.* 23 10495–10502. 10.1523/JNEUROSCI.23-33-10495.2003 14627633 PMC6740930

[B122] Selcho M. (2024). Octopamine in the mushroom body circuitry for learning and memory. *Learn. Mem.* 31:a053839. 10.1101/lm.053839.123 38862169 PMC11199948

[B123] Selcho M. Pauls D. El Jundi B. Stocker R. F. Thum A. S. (2012). The role of octopamine and tyramine in *Drosophila* larval locomotion. *J. Comp. Neurol.* 520 3764–3785. 10.1002/cne.23152 22627970

[B124] Selcho M. Pauls D. Han K. A. Stocker R. F. Thum A. S. (2009). The role of dopamine in *Drosophila* larval classical olfactory conditioning. *PLoS One* 4:e5897. 10.1371/journal.pone.0005897 19521527 PMC2690826

[B125] Selcho M. Pauls D. Huser A. Stocker R. F. Thum A. S. (2014). Characterization of the octopaminergic and tyraminergic neurons in the central brain of *Drosophila* larvae. *J. Comp. Neurol.* 522 3485–3500. 10.1002/cne.23616 24752702

[B126] Sitaraman D. Zars M. Zars T. (2010). Place memory formation in Drosophila is independent of proper octopamine signaling. *J. Comp. Physiol. A Neuroethol. Sens. Neural Behav. Physiol.* 196 299–305. 10.1007/s00359-010-0517-5 20228998

[B127] Stowers R. S. (2011). An efficient method for recombineering GAL4 and QF drivers. *Fly* 5 371–378. 10.4161/fly.5.4.17560 21857163 PMC3266080

[B128] Sujkowski A. Wessells R. (2018). Using drosophila to understand biochemical and behavioral responses to exercise. *Exerc. Sport Sci. Rev.* 46 112–120. 10.1249/JES.0000000000000139 29346165 PMC5856617

[B129] Sujkowski A. Ramesh D. Brockmann A. Wessells R. (2017). Octopamine drives endurance exercise adaptations in drosophila. *Cell. Rep.* 21 1809–1823. 10.1016/j.celrep.2017.10.065 29141215 PMC5693351

[B130] Suver M. P. Mamiya A. Dickinson M. H. (2012). Octopamine neurons mediate flight-induced modulation of visual processing in Drosophila. *Curr. Biol.* 22 2294–2302. 10.1016/j.cub.2012.10.034 23142045

[B131] Szuperak M. Churgin M. A. Borja A. J. Raizen D. M. Fang-Yen C. Kayser M. S. (2018). A sleep state in Drosophila larvae required for neural stem cell proliferation. *eLife* 7:e33220. 10.7554/eLife.33220 29424688 PMC5834245

[B132] Tomancak P. Beaton A. Weiszmann R. Kwan E. Shu S. Q. Lewis S. E. et al. (2002). Systematic determination of patterns of gene expression during *Drosophila* embryogenesis. *Genome Biol.* 3:dev175265. 10.1186/gb-2002-3-12-research0088 12537577 PMC151190

[B133] Tomancak P. Berman B. P. Beaton A. Weiszmann R. Kwan E. Hartenstein V. et al. (2007). Global analysis of patterns of gene expression during *Drosophila* embryogenesis. *Genome Biol.* 8:R145. 10.1186/gb-2007-8-7-r145 17645804 PMC2323238

[B134] Tsubouchi A. Caldwell J. C. Tracey W. D. (2012). Dendritic filopodia, ripped pocket, NOMPC, and NMDARs contribute to the sense of touch in *Drosophila* larvae. *Curr. Biol.* 22 2124–2134. 10.1016/j.cub.2012.09.019 23103192 PMC3511824

[B135] van Breugel F. Suver M. P. Dickinson M. H. (2014). Octopaminergic modulation of the visual flight speed regulator of Drosophila. *J. Exp. Biol.* 217 1737–1744. 10.1242/jeb.098665 24526725

[B136] Venken K. J. Schulze K. L. Haelterman N. A. Pan H. He Y. Evans-Holm M. et al. (2011). MiMIC: A highly versatile transposon insertion resource for engineering *Drosophila melanogaster* genes. *Nat. Methods* 8 737–743. 10.1038/nmeth.1662 21985007 PMC3191940

[B137] Wang Q. P. Lin Y. Q. Zhang L. Wilson Y. A. Oyston L. J. Cotterell J. et al. (2016). Sucralose promotes food intake through NPY and a neuronal fasting response. *Cell. Metab.* 24 75–90. 10.1016/j.cmet.2016.06.010 27411010

[B138] Wang Y. De Backer J. F. Muria A. Abe A. Saito K. Holvoet H. et al. (2026). A bidirectional brain-fat body axis for pathogen avoidance. *Neuron* 10.1016/j.neuron.2026.03.026 41997146 [Epub ahead of print].

[B139] Watanabe K. Chiu H. Pfeiffer B. D. Wong A. M. Hoopfer E. D. Rubin G. M. et al. (2017). A circuit node that integrates convergent input from neuromodulatory and social behavior-promoting neurons to control aggression in *Drosophila*. *Neuron* 95 1112–1128.e1117. 10.1016/j.neuron.2017.08.017 28858617 PMC5588916

[B140] Weber D. Richter V. Rohwedder A. Großjohann A. Thum A. S. (2023a). The analysis of aversive olfactory-taste learning and memory in Drosophila Larvae. *Cold Spring Harb. Protoc.* 2023:108050-pdb prot. 10.1101/pdb.prot108050 36180215

[B141] Weber D. Richter V. Rohwedder A. Großjohann A. Thum A. S. (2023b). Learning and memory in drosophila larvae. *Cold Spring Harb. Protoc.* 2023:107863-pdb top. 10.1101/pdb.top107863 36180213

[B142] Wipfler B. Schneeberg K. Loffler A. Hunefeld F. Meier R. Beutel R. G. (2013). The skeletomuscular system of the larva of *Drosophila melanogaster* (Drosophilidae, Diptera): A contribution to the morphology of a model organism. *Arthropod. Struct. Dev.* 42 47–68. 10.1016/j.asd.2012.09.005 23010508

[B143] Wu H. Eno S. Jinnai K. Abe A. Saito K. Maekawa Y. et al. (2025). Octopamine signaling regulates the intracellular pattern of the presynaptic active zone scaffold within Drosophila mushroom body neurons. *PLoS Biol.* 23:e3003449. 10.1371/journal.pbio.3003449 41129552 PMC12626334

[B144] Yang C. H. Shih M. F. Chang C. C. Chiang M. H. Shih H. W. Tsai Y. L. et al. (2016). Additive expression of consolidated memory through *Drosophila* mushroom body subsets. *PLoS Genet.* 12:e1006061. 10.1371/journal.pgen.1006061 27195782 PMC4873240

[B145] Yang Z. Yu Y. Zhang V. Tian Y. Qi W. Wang L. (2015). Octopamine mediates starvation-induced hyperactivity in adult *Drosophila*. *Proc. Natl. Acad. Sci. U S A.* 112 5219–5224. 10.1073/pnas.1417838112 25848004 PMC4413307

[B146] Yarali A. Gerber B. (2010). A neurogenetic dissociation between punishment-, reward-, and relief-learning in *Drosophila*. *Front. Behav. Neurosci.* 4:189. 10.3389/fnbeh.2010.00189 21206762 PMC3013555

[B147] Yoshinari Y. Ameku T. Kondo S. Tanimoto H. Kuraishi T. Shimada-Niwa Y. et al. (2020). Neuronal octopamine signaling regulates mating-induced germline stem cell increase in female Drosophila melanogaster. *Elife* 9:e57101. 10.7554/eLife.57101 33077027 PMC7591258

[B148] Youn H. Kirkhart C. Chia J. Scott K. (2018). A subset of octopaminergic neurons that promotes feeding initiation in Drosophila melanogaster. *PLoS One* 13:e0198362. 10.1371/journal.pone.0198362 29949586 PMC6021039

[B149] Yu Y. Huang R. Ye J. Zhang V. Wu C. Cheng G. et al. (2016). Regulation of starvation-induced hyperactivity by insulin and glucagon signaling in adult *Drosophila*. *eLife* 5:e15693. 10.7554/eLife.15693 27612383 PMC5042652

[B150] Zarin A. A. Mark B. Cardona A. Litwin-Kumar A. Doe C. Q. (2019). A multilayer circuit architecture for the generation of distinct locomotor behaviors in *Drosophila*. *eLife* 8:e51781. 10.7554/eLife.51781 31868582 PMC6994239

[B151] Zhang T. Branch A. Shen P. (2013). Octopamine-mediated circuit mechanism underlying controlled appetite for palatable food in Drosophila. *Proc. Natl. Acad. Sci. U S A.* 110 15431–15436. 10.1073/pnas.1308816110 24003139 PMC3780881

[B152] Zhao Z. Zhao X. He T. Wu X. Lv P. Zhu A. J. et al. (2021). Epigenetic regulator Stuxnet modulates octopamine effect on sleep through a Stuxnet-Polycomb-Octbeta2R cascade. *EMBO Rep.* 22:e47910. 10.15252/embr.201947910 33410264 PMC7857429

[B153] Zhou C. Huang H. Kim S. M. Lin H. Meng X. Han K. A. et al. (2012). Molecular genetic analysis of sexual rejection: Roles of octopamine and its receptor OAMB in *Drosophila* courtship conditioning. *J. Neurosci.* 32 14281–14287. 10.1523/JNEUROSCI.0517-12.2012 23055498 PMC3670963

[B154] Zhou C. Rao Y. Rao Y. (2008). A subset of octopaminergic neurons are important for *Drosophila* aggression. *Nat. Neurosci.* 11 1059–1067. 10.1038/nn.2164 19160504

