# Supplementary material for: Octopamine receptors in Drosophila larvae: expression, development, and behavior
Source: Front Mol Neurosci. 2026 Sep 3;19:1876842. doi: 10.3389/fnmol.2026.1876842 (PMC13584740; doi:10.3389/fnmol.2026.1876842)
Supplement: Supplementary file 1 [file Data_Sheet_1.docx]

**Supplementary Figure 1: Trojan *Octβ2R* labeling throughout the body of third-instar larva.** Using native fluorescence, the labeling of Octβ2R (green via mCherry) and neuronal Synaptobrevin, as a reference channel (magenta via GFP), is shown. **(A)** Ventral view of the larva shows ventral longitudinal (muscle 6, 7), ventral oblique (muscle 15, 16, 17), and ventral acute (muscle 29) muscles. **(B)** Dorsal view of the terminal shows dorsal transverse muscle 48. **(C)** Octβ2R signal was identified in at least one sensory neuron in the terminal organ (arrows). Additional muscles are labelled for orientation. A2/3/7/8/9: abdominal segment 2/3/7/8/9. Scale bar: 100 µm.

**Supplementary Figure 2: Trojan Exon octopamine receptor labeling throughout the body of first-instar larvae.** **(A)-(F)** Using native fluorescence, the labeling of each octopamine receptor (green via mCherry) and neuronal Synaptobrevin, as a reference channel (magenta via GFP), is shown. A4/5/8/9: abdominal segment 4/5/8/9; PCE: pseudocephalon..Scale bar: 100 µm.

**Supplementary Figure 3: Summary of Trojan Exon octopamine receptor labeling throughout the body of first-instar larvae.** The table summarizes the labeling in the first instar larva (see Supplementary Figure 2). ChOs: Chordotonal organ; CNS: Central nervous system; DAs: Dendritic arborization neurons; DLM: dorsal longitudinal muscle; DO: Dorsal organ; DOM: dorsal oblique muscle; DPS: Dorsal pharyngeal sensilla; ESs: External sense neurons; LTM: lateral transverse muscle; PCE: pseudocephalon; PNS: Peripheral nervous system; SSO: Spiracle sense organ; TO: Terminal organ; TSCs: Terminal sense organ; VLM: ventral longitudinal muscle.

**Supplementary Figure 4: Sequencing result of the amplified Trojan Exon insert location in Trojan *Tβh*.** The schematic shows the location of the primer according to the MI04010 docking site, which was used to insert the Trojan Exon. One primer is upstream of the docking site; the second primer is downstream. Both are located in the gene region of *Tβh*. Below, the sequenced amplicon is compared with the gene region of *Tβh*. The sequenced amplicon has a size of 296 bp, a bit shorter than the observed product in the gel picture (Figure 7A_I_), which is due to the sequencing process. The upper row is the reference sequence (Ref. seq), and the lower row is the sequenced amplicon (Trojan TBh). Red: primer; green: MI04010 docking site; Dots show same bases, dashes show missing bases, and asterisks show dissimilar bases.

**Supplementary Figure 5: Sequencing results of amplified Trojan Exon insert location in Trojan *Oamb.*** **(A)** Schematic of the PCR design used for verification of the insert location of the Trojan Exon in *Oamb*. Red arrows = primer **(B)** Verification of the insertion site of the Trojan Exon: the sequencing results of the amplified product depicted in Figure 7B_I_ (*Oamb*) is shown. Reference sequence (Ref. seq; upper row) starts and ends with the primer (red arrows in A) used during the PCR. The first primer is located in an Intron (grey), which is upstream of the Trojan Exon, followed by the *Minos* inverted repeat (orange) and the Trojan Exon itself (partially covered with the attB (red), splice acceptor (violet), linker (yellow), T2A (blue), and partial Gal4 (green)). The second primer is located in the Gal4 of the Trojan Exon; hence, the Gal4 is sequenced only partially. The reference sequence shows only the region that is amplified using the selected primer, not the entire Intron and Trojan Exon. The lower row is the sequenced amplicon (Trojan Exon octopamine receptor line). Grey: Intron; orange: *Minos* inverted repeat; red: attB; violet: splice acceptor; yellow: linker; blue: T2A; green: Gal4; Bold letters: MiMIC docking site; Dots show same bases, dashes show missing bases, asterisks show dissimilar bases; K: Guanine/Thymine; N: unclear nucleotide; R: Guanine/Adenine; W: Adenine/Thymine.

**Supplementary Figure 6: Sequencing results of amplified Trojan Exon insert location in Trojan *Octα2R.*** Verification of the insertion site of the Trojan Exon: the sequencing results of the amplified product depicted in Figure 7C_I_ (*Octα2R*) is shown. The Reference sequence (Ref. seq; upper row) starts and ends with the primer used during the PCR (a schematic is shown in Supplementary Figure 5A). The first primer is located in an Intron (grey), which is upstream of the Trojan Exon, followed by the *Minos* inverted repeat (orange) and the Trojan Exon itself (partially covered with the attB (red), splice acceptor (violet), linker (yellow), T2A (blue), and partial Gal4 (green)). The second primer is located in the Gal4 of the Trojan Exon; hence, the Gal4 is sequenced only partially. The reference sequence shows only the region that is amplified using the selected primer, not the entire Intron and Trojan Exon. The lower row is the sequenced amplicon (Trojan Exon octopamine receptor line). Grey: Intron; orange: *Minos* inverted repeat; red: attB; violet: splice acceptor; yellow: linker; blue: T2A; green: Gal4; Bold letters: MiMIC docking site; Dots show same bases, dashes show missing bases, asterisks show dissimilar bases; K: Guanine/Thymine; N: unclear nucleotide; R: Guanine/Adenine; W: Adenine/Thymine.

**Supplementary Figure 7: Sequencing results of amplified Trojan Exon insert location in Trojan *Octβ1R.*** Verification of the insertion site of the Trojan Exon: the sequencing results of the amplified product depicted in Figure 7D_I_ (*Octβ1R*) is shown. The Reference sequence (Ref. seq; upper row) starts and ends with the primer used during the PCR (a schematic is shown in Supplementary Figure 5A). The first primer is located in an Intron (grey), which is upstream of the Trojan Exon, followed by the *Minos* inverted repeat (orange) and the Trojan Exon itself (partially covered with the attB (red), splice acceptor (violet), linker (yellow), T2A (blue), and partial Gal4 (green)). The second primer is located in the Gal4 of the Trojan Exon; hence, the Gal4 is sequenced only partially. The reference sequence shows only the region that is amplified using the selected primer, not the entire Intron and Trojan Exon. The lower row is the sequenced amplicon (Trojan Exon octopamine receptor line). Grey: Intron; orange: *Minos* inverted repeat; red: attB; violet: splice acceptor; yellow: linker; blue: T2A; green: Gal4; Bold letters: MiMIC docking site; Dots show same bases, dashes show missing bases, asterisks show dissimilar bases; K: Guanine/Thymine; N: unclear nucleotide; R: Guanine/Adenine; W: Adenine/Thymine.

**Supplementary Figure 8: Sequencing results of amplified Trojan Exon insert location in Trojan *Octβ3R.*** Verification of the insertion site of the Trojan Exon: the sequencing results of the amplified product depicted in Figure 7F_I_ (*Octβ3R*) is shown. The Reference sequence (Ref. seq; upper row) starts and ends with the primer used during the PCR (a schematic is shown in Supplementary Figure 5A). The first primer is located in an Intron (grey), which is upstream of the Trojan Exon, followed by the *Minos* inverted repeat (orange) and the Trojan Exon itself (partially covered with the attB (red), splice acceptor (violet), linker (yellow), T2A (blue), and partial Gal4 (green)). The second primer is located in the Gal4 of the Trojan Exon; hence, the Gal4 is sequenced only partially. The reference sequence shows only the region that is amplified using the selected primer, not the entire Intron and Trojan Exon. The lower row is the sequenced amplicon (Trojan Exon octopamine receptor line). Grey: Intron; orange: *Minos* inverted repeat; red: attB; violet: splice acceptor; yellow: linker; blue: T2A; green: Gal4; Bold letters: MiMIC docking site; Dots show same bases, dashes show missing bases, asterisks show dissimilar bases; K: Guanine/Thymine; N: unclear nucleotide; R: Guanine/Adenine; W: Adenine/Thymine.

**Supplementary Figure 9: Sequencing results of amplified Trojan Exon insert location in Trojan *Oct-TyrR*.** Verification of the insertion site of the Trojan Exon: the sequencing results of the amplified product depicted in Figure 7G_I_ (*Oct-TyrR*) is shown. The Reference sequence (Ref. seq; upper row) starts and ends with the primer used during the PCR (a schematic is shown in Supplementary Figure 5A). The first primer is located in an Intron (grey), which is upstream of the Trojan Exon, followed by the *Minos* inverted repeat (orange) and the Trojan Exon itself (partially covered with the attB (red), splice acceptor (violet), linker (yellow), T2A (blue), and partial Gal4 (green)). The second primer is located in the Gal4 of the Trojan Exon; hence, the Gal4 is sequenced only partially. The reference sequence shows only the region that is amplified using the selected primer, not the entire Intron and Trojan Exon. The lower row is the sequenced amplicon (Trojan Exon octopamine receptor line). Grey: Intron; orange: *Minos* inverted repeat; red: attB; violet: splice acceptor; yellow: linker; blue: T2A; green: Gal4; Bold letters: MiMIC docking site; Dots show same bases, dashes show missing bases, asterisks show dissimilar bases; K: Guanine/Thymine; N: unclear nucleotide; R: Guanine/Adenine; W: Adenine/Thymine.

**Supplementary Figure 10: Sequencing result of the 5 ′ additional parts of Trojan Exon in Trojan *Octβ2R*.** During PCR analysis, a larger fragment than expected was observed (expected: 1072 bp, observed: ~ 3000 bp). Here, the sequencing result of the amplified product depicted in Figure 7E_I_ is shown. Due to the large size, the amplicon had to be sequenced in two steps as depicted in schematic **(A)**: first, with a primer located in the genomic region of *Octβ2R* (red arrow, sequencing result shown in B) and second, with a primer located in the Trojan Exon (blue arrow, sequencing result shown in Supplementary Figure 11). **(B)** The upper row is the reference sequence (Ref. seq) consisting of Intron 6 (grey), that is upstream of the Trojan Exon, the *Minos* inverted repeat (orange), and the Trojan Exon itself (partially covered with the attB (red), splice acceptor (violet), linker (yellow), T2A (blue), and partial Gal4 (green)). The reference sequence shows only the region that is amplified using the selected primer, not the entire Intron 6 and Trojan Exon. The upper row is the reference sequence (Ref. seq), and the lower row is the sequenced amplicon (Trojan). Grey: Intron 6; orange: *Minos* inverted repeat; red: attB; violet: splice acceptor; yellow: linker; blue: T2A; green: Gal4; Bold letters: MiMIC docking site; Dots show same bases, dashes show missing bases, asterisks show dissimilar bases; W: Adenine/Thymine.

**Supplementary Figure 11: Sequencing result of 5 ′ additional parts of Trojan Exon in Trojan *Octβ2R*.** During PCR analysis, a larger fragment than expected was observed (expected: 1072 bp, observed: ~ 3000 bp). Here, the sequencing result of the amplified product depicted in Figure 7E_I_ is shown. Due to the large size, the amplicon had to be sequenced in two steps as depicted in Supplementary Figure 10A: first, with a primer located in the genomic region of *Octβ2R* (red arrow, sequencing result shown in Supplementary Figure 10B) and second, with a primer located in the Trojan Exon (blue arrow, sequencing result shown here). The upper row is the reference sequence (Ref. seq) consisting of Intron 6 (grey), that is upstream of the Trojan Exon, the *Minos* inverted repeat (orange), and the Trojan Exon itself (partially covered with the attB (red), splice acceptor (violet), linker (yellow), T2A (blue), and partial Gal4 (green)). The reference sequence shows only the region that is amplified using the selected primer, not the entire Intron 6 and Trojan Exon. The upper row is the reference sequence (Ref. seq), and the lower row is the sequenced amplicon (Trojan). Grey: Intron 6; orange: *Minos* inverted repeat; red: attB; violet: splice acceptor; yellow: linker; blue: T2A; green: Gal4; Bold letters: MiMIC docking site; Dots show same bases, dashes show missing bases, asterisks show dissimilar bases; W: Adenine/Thymine.

**Supplementary Figure 12: Sequencing result of the 3 ′ additional parts of Trojan Exon in Trojan *Octβ2R*.** During PCR analysis, a larger fragment than expected was observed (expected: 1366 bp, observed: ~1500 bp). Here, the sequencing results of the amplified product depicted in Figure 7E_II_ is shown. For sequencing, a primer located in the *Octβ2R* genomic region was selected. The upper row is the reference sequence (Ref. seq) consisting of Intron 6 (grey), which is downstream of the Trojan Exon, the *Minos* inverted repeat (orange), and the Trojan Exon itself (partially covered with the attB (red), splice donor (violet), Hsp70 polyA (brown), and partial Gal4 (green)). The reference sequence shows only the region that is amplified using the selected primer, not the entire Intron 6 and Trojan Exon. The upper row is the reference sequence (Ref. seq), and the lower row is the sequenced amplicon (Trojan). Grey: Intron 6; orange: *Minos* inverted repeat; red: attB; violet: splice donor; brown: Hsp70 polyA; green: Gal4; Dots show same bases, dashes show missing bases, asterisks show dissimilar bases; N: unclear nucleotide.

**Supplementary Figure 13: mRNA analysis of Trojan** ***Octβ2R* and *Oct-TyrR*.** Qualitative RT-PCR of Trojan *Octβ2R* showed that additional sequences that were found at the genomic level (Figure 7E_I_ and E_II_) were not included in the Trojan *Octβ2R* mRNA. In **(A_I_)**, a schematic shows the location of the unknown sequence (green) right upstream of the Trojan Exon (blue) in the genome (schematic not to scale). Below, a schematic shows the location of the primer used during RT-PCR (red arrows). The size of the *Octβ2R* mRNA product in the Trojan *Octβ2R* was between 500 and 600 bp. The expected minimal size was 532 bp, which includes the region of the upstream Exon and the Trojan Exon. Product size would have been larger than the minimal 532 bp if additional sequences were included in the mRNA. Sequencing confirmed that no additional unknown sequences are included in the Trojan *Octβ2R* mRNA (Supplementary Figure 14). **(C)** Moreover, quantitative real-time RT-PCR showed that only a fraction (~6 %) of *Octβ2R* mRNA is present in the Trojan *Octβ2R* (normalized to housekeeping gene *Rpl32* and compared to *y^1^w**). In **(A)** the binding site of the TaqMan probe is depicted (yellow): spanning Exon 1/2 and 3, which is downstream of the inserted Trojan Exon in Intron 6. **(B)** and **(C)** Due to the insertion site of the Trojan Exon in *Oct-TyrR* (5´UTR), quantitative real-time RT-PCR was performed to measure transcript levels of *Oct-TyrR*. **(B)** shows the binding site of the TaqMan probe in the *Oct-TyrR* transcripts (yellow), which is downstream of the Trojan Exon insert location. **(C)** *Oct-TyrR* mRNA levels were undeterminable in the Trojan *Oct-TyrR* during quantitative real-time RT-PCR. Orange: predicted conserved domains for *Octβ2R* and *Oct-TyrR* (see Supplementary Table 8); **** = p < 0.0001.

**Supplementary Figure 14: Sequencing result of Trojan *Octβ2R* mRNA to analyze additional regions.** Trojan *Octβ2R* mRNA was analyzed regarding the additional sequences that were found at the genomic level. Here, the sequencing result of the amplified product depicted in Supplementary Figure 13A_I_ is shown. The upper row is the reference sequence (Ref. seq) consisting of Exon 7 (red) that is upstream of the Trojan Exon and the Trojan Exon itself (partially covered with the linker (yellow), T2A (blue), and partial Gal4 (green)). The reference sequence shows only the region that is amplified using the selected primer (not the entire Exon 7) and the Trojan Exon. The sequenced amplicon has a size of 470 bp, a bit shorter than the observed product in the gel picture (Supplementary Figure 13A_I_), which is due to the sequencing process. The upper row is the reference sequence (Ref. seq), and the lower row is the sequenced amplicon of Trojan *Octβ2R* mRNA (OctB2R). Red: Exon 7; yellow: linker (belongs to Trojan Exon); blue: T2A; green: Gal4; Dots show the same bases, dashes show missing bases, and asterisks show dissimilar bases.
